# In Situ Electrochemical Monitoring and Removal of Oxide Ions in FLiBe Molten Salts

**DOI:** 10.3390/ma19183868

**Published:** 2026-09-11

**Authors:** Xiaoqin Fu, Lei Wang, Yingjie Li, Xu Li, Zhuyao Li, Yuyang Huang, Haiying Fu, Qiang Dou, Hao Peng

**Affiliations:** 1 Shanghai Institute of Applied Physics, Chinese Academy of Sciences, Shanghai 201800, China; 2 University of Chinese Academy of Sciences, Beijing 100049, China; 3 School of Chemistry and Materials Science, East China University of Technology, Nanchang 330013, China; 4 Wuwei Institute of Advanced Energy, Wuwei 733000, China

**Keywords:** molten salt reactors (MSRs), fluoride molten salts, square wave voltammetry (SWV), O^2−^ concentration, electrochemical behavior of O^2−^, potentiostatic electrolysis, O^2−^ removal

## Abstract

In this work, cyclic voltammetry, square wave voltammetry, and chronopotentiometry were employed to systematically investigate the electrochemical behavior of O^2−^ in LiF–BeF_2_ (66.7: 33.3 mol%) (FLiBe) molten salt on a gold electrode. Comparative analysis demonstrated that square wave voltammetry is more suitable than cyclic voltammetry for studying the electrochemical behavior of O^2−^. The square wave voltammetry results confirmed that O^2−^ is oxidized to O_2_, with the electrode reaction controlled by ion diffusion. chronopotentiometry confirmed that the electrochemical behavior of O^2−^ obeyed the Sand law. The diffusion coefficient of O^2−^ in FLiBe by chronopotentiometry followed Arrhenius’ law. Then, a well-fitted linear relationship between O^2−^ concentration and square wave voltammetry peak current density was established, enabling rapid determination and real-time monitoring of oxide content in an unknown FLiBe molten salt. Furthermore, the electrochemical deoxygenation of FLiBe was performed using the potentiostatic electrolysis technique, and a satisfactory removal efficiency of O^2−^ was achieved. This study presents an in situ electrochemical quantitative analysis method to monitor the O^2−^ concentration in FLiBe, overcoming the drawback of the long testing cycle in conventional chemical analysis. Meanwhile, this study provides new technical support for the deoxygenation and purification of the fuel salt in molten salt reactors.

## 1. Introduction

Fission nuclear energy, due to its high energy density, low carbon emissions, and potential for sustainable development, has become a candidate for the main clean energy source in the coming decades [1]. The molten salt reactor (MSR) has been selected as one of the six advanced fourth-generation nuclear reactors [2]. MSR has unique advantages such as inherent safety, flexible fuel-cycle characteristics, efficient transmutation of actinide elements, high thermal power density, easy realization of small-scale modularization, and less radioactive waste [3,4,5]. As the only fourth-generation reactor that uses liquid fuel, MSR adopts fluoride molten salt systems as both coolant and fuel salt due to their excellent nuclear physical properties, outstanding thermohydraulic properties, and good chemical stability [6,7].

In the Molten Salt Reactor Experiment (MSRE) and Molten Salt Breeder Reactor (MSBR) programs by Oak Ridge National Laboratory (ORNL), LiF–BeF_2_–ZrF_4_–UF_4_ (65–29.1–5–0.9 mol%) and LiF–BeF_2_–ThF_4_–UF_4_ (71.7–16–12–0.3 mol%) were used as primary fuel systems, respectively [8,9]. Recently, a 2 MWt liquid-fuel thorium molten salt experimental reactor (TMSR-LF1) led by Shanghai Institute of Applied Physics, Chinese Academy of Sciences (SINAP@CAS), has successfully realized thorium–uranium fuel conversion in the LiF–BeF_2_–ZrF_4_–ThF_4_–UF_4_ system [10]. Therefore, LiF–BeF_2_ (FLiBe) is the most important fuel carrier medium and coolant for MSR.

Nevertheless, fluoride salt raw materials readily absorb trace moisture from air even under low-humidity conditions [11]. The adsorbed water undergoes hydrolysis at high temperatures (Equation (1)) [12], yielding reactive oxide ions (O^2−^). In addition, the corrosion of the oxide passivation layer on the inner surface of the structural material also leads to an increase in the O^2−^ content in the molten salt. Meanwhile, the active oxide can be produced through the neutron irradiation of fuel salt and the alpha-ray irradiation of carbon moderator [13]. Furthermore, the fatigue damage of loop or welding parts may bring in air and oxygen as well [14,15]. Therefore, the source of impurity O^2−^ in MSR is extensive, and O^2−^ has become one of the most typical impurities in fluoride molten salts.

(1) $$H_{2}O+2F^{-}\to \;2\mathrm{HF}+{\;O}^{2-}$$

O^2−^ exhibits extremely high chemical activity in molten fluoride salts. Metal ions in the molten salts are highly sensitive to O^2−^, and the interactions between metal ions and O^2−^ occur at high temperatures to form diverse oxide and oxyfluoride species, e.g., the fuel UF_4_ and ThF_4_ tends to combine with O^2−^ to form insoluble oxygen-containing compounds, such as oxides (UO_2_, ThO_2_) and oxyfluorides ((UOF_2_)*_n_* and (ThOF_2_)*_n_*). These precipitates feature low solubility, high melting point, and high density compared with the molten salt medium. Continuous accumulation of nuclear fuel precipitates not only causes loss of soluble fuel, but also blocks pipelines of fuel circuits, hinders mass transfer, and disrupts the thermal-hydraulic characteristics that enable the reactor to operate stably. More severely, localized enrichment of nuclear fuel precipitates may generate overheating zones, posing severe threats to reactor safety [16]. Accordingly, strict control of oxide impurity content in fluoride salts is essential.

ORNL proposed that bubbling treatment with HF/H_2_ mixed gas serves as an effective approach to eliminate O^2−^ from fluoride molten salts in MSRE and MSBR programs. Specifically, HF/H_2_ mixed gas with a molar ratio of 1:10 is sparged into molten salts. O^2−^ reacts with HF to generate water vapor, thereby reducing the oxide content, as illustrated in Equation (2). Via this method, ORNL successfully controlled the oxide impurity content below 700 ppm in LiF–BeF_2_–ZrF_4_–UF_4_ molten salt (65–29.1–5–0.9 mol%) at 650 °C [17], and D. A. Petti et al. [18] further reduced the oxide content of FLiBe to 560 ppm. V. Afonichkin et al. [19] adopted ammonium bifluoride (NH_4_HF_2_) to replace HF for the purification of the LiF–ThF_4_–UF_4_ system and achieved favorable deoxidation performance, and the corresponding reaction is presented in Equation (3). Although both HF/H_2_ bubbling and ammonium bifluoride methods can effectively reduce oxide impurities, their deoxidation capacity is restricted by chemical equilibrium with inherent oxide removal limitations. Moreover, these techniques fail to eliminate trace oxide generated during reactor operation. Consequently, oxide impurities cannot be completely removed from fluoride molten salts. Since O^2−^ cannot be avoided, it is necessary to establish the oxide content standard for the MSR. The maximum oxide content allowed in the TMSR-LF1 fuel salt is 200 ppm.

(2) $$O^{2-}+\;2\mathrm{HF}\;\to \;H_{2}O↑+\;2F^{-}$$

(3) $${2O}^{2-}+\;4{\mathrm{NH}}_{4}{\mathrm{HF}}_{2}\;\to \;{2H}_{2}O↑+\;4{\mathrm{NH}}_{4}F↑+\;4F^{-}$$

The precise analysis of oxide content in fluoride molten salts is of great significance for the safe operation of MSRs. ORNL adopted the inert gas fusion method [20], HF–H_2_ method [21], and the KBrF_4_ oxidation method [22] for oxide quantification. In the inert gas fusion method, molten fluoride specimens are heated under a high-temperature inert atmosphere, and oxide impurities are converted into carbon dioxide and water vapor, whose contents are quantitatively measured via an infrared detector. In the HF–H_2_ method, oxide ions react with anhydrous HF and transform into water vapor, and oxide content is calculated according to the generated vapor amount. The KBrF_4_ oxidation method relies on a redox reaction between molten fluoride salt and KBrF_4_ at 450 °C, where O^2−^ is oxidized into oxygen gas, and oxide concentration is determined by gas pressure measurement. In recent years, LECO Corporation has developed oxide analyzers based on inert-atmosphere fusion and carbothermal reduction principles. The analyzer has a limit of detection of 0.01 μg for oxide, with analytical accuracy of 0.025 ppm or 0.5% RSD, which satisfies the analytical requirements of oxide content in fluoride salt systems.

However, all the above-mentioned O^2−^ concentration detection methods are based on the online sampling–offline analysis mode, which has shortcomings such as time-consuming sampling, complicated sample preparation, and a long testing cycle. In addition, contamination by impurities during sample storage and transportation may interfere with test results. Moreover, there are issues such as beryllium leakage and high operational risks, making it difficult to meet the real-time monitoring and cleaning analysis requirements of trace oxide impurities in fluoride salt systems. Therefore, it is urgent to develop an in situ analysis method to achieve rapid determination of O^2−^ concentration.

An electrochemical method has been proposed for the investigation of electroactive species because of its quick response and in situ monitoring. Voltammetry has been established as an accurate, in situ technique for measuring the concentration of ions in high-temperature molten salts. Relevant studies have been extensively conducted in chloride and fluoride molten salt systems. In the typical LiCl–KCl eutectic chloride melt, Serp et al. [23] studied the redox behavior of plutonium ions, while Shirai et al. [24] studied the redox and deposition behaviors of uranium and plutonium ions in LiCl–KCl eutectic melts, and determined their relevant thermodynamic parameters. Fang et al. [25] also analyzed the electrode kinetics of UCl_3_ in LiCl–KCl melt at 773 K via cyclic voltammetry (CV) and square wave voltammetry (SWV), acquiring key uranium kinetic parameters, validating the reliability of voltammetry for actinide speciation analysis in chloride molten salts. For fluoride molten salts, Peng et al. [12] characterized the redox and diffusion behaviors of uranium species in FLiNaK salt via CV and SWV; an electrochemical quantitative analysis method for U(IV) was then established, and the concentration of U(IV) was monitored. Furthermore, Peng et al. [26] also utilized SWV to conduct an analysis of the concentration and stability of chromium species in the FLiNaK (LiF–NaF–KF, 46.5–11.5–42 mol%) molten salt. Zhao et al. [27] investigated the redox characteristics and dissolution stability of actinide ions in FLiBe fuel salt. These studies demonstrate that voltammetric techniques are efficient and reliable for qualitative and quantitative analysis of electroactive species in molten salts.

In recent years, electrochemical detection has attained preliminary progress in oxide monitoring of high-temperature molten salts. Massot et al. [28] investigated the electrochemical behaviors of O^2−^ in binary LiF–NaF molten salt via CV, SWV, and chronopotentiometry (CP). They found that SWV and CP are more suitable for O^2−^ analysis than CV. Unfortunately, their research did not take into account other oxide species in molten salt and did not calibrate the actual concentration of free O^2−^. Shen et al. [29] systematically explored the electrochemical characteristics of O^2−^ in the FLiNaK molten salt at 600 °C using SWV, and a linear quantitative correlation between the oxidation peak current and O^2−^ concentration was established. However, their research lacks comparisons with other electrochemical analysis methods. Choi et al. [30] proposed that SWV could be used to monitor O^2−^ concentration during the electrochemical reduction of uranium oxide in molten Li_2_O–LiCl. Manning et al. [31] determined the electrochemical behavior of O^2−^ and O_2_^2−^ in LiF–BeF_2_–ZrF_4_ and LiF–BeF_2_–ThF_4_ melts by CV, and proved that the oxidation of the two ions was controlled by ion diffusion.

The above findings demonstrate the potential of SWV for O^2−^ detection in molten salts. However, existing studies have notable limitations: most investigations focus on non-beryllium fluoride systems, which are not fully applicable to practical MSR fuel salts. Moreover, previous works lack systematic quantitative calibration, multi-method comparative analysis, and O^2−^ impurity removal technology. To date, no complete studies have reported the electrochemical behavior, precise quantification, and electrolytic elimination of O^2−^ in FLiBe systems, restricting the accurate monitoring and control of oxide impurities for MSR operation.

Against this background, this work systematically investigates O^2−^ electrochemical behaviors in FLiBe salt via CV, SWV, and CP for the first time and establishes a high-precision in situ SWV quantitative method for trace O^2−^. Furthermore, potentiostatic electrolysis is adopted to achieve efficient removal of O^2−^ impurities. The developed technical system can stabilize oxide content within the safe threshold of TMSR-LF1, eliminate reactor safety risks induced by oxide precipitation, and provide a reliable technical reference for the safe operation and engineering application of MSRs.

In this work, CV, SWV, and CP were employed to systematically study the electrochemical behavior of O^2−^ in FLiBe molten salt. For the first time, the key electrochemical parameters of O^2−^ in the FLiBe system were obtained. Then, the SWV method was employed to establish a quantitative electroanalytical method for O^2−^. Based on this, the O^2−^ content in the unknown FLiBe molten salt was rapidly determined and monitored in real time. Finally, the constant potential electrolysis technique was employed to effectively remove O^2−^ from the FLiBe system.

## 2. Experimental

### 2.1. Experimental Technical Route

**Figure 1:**
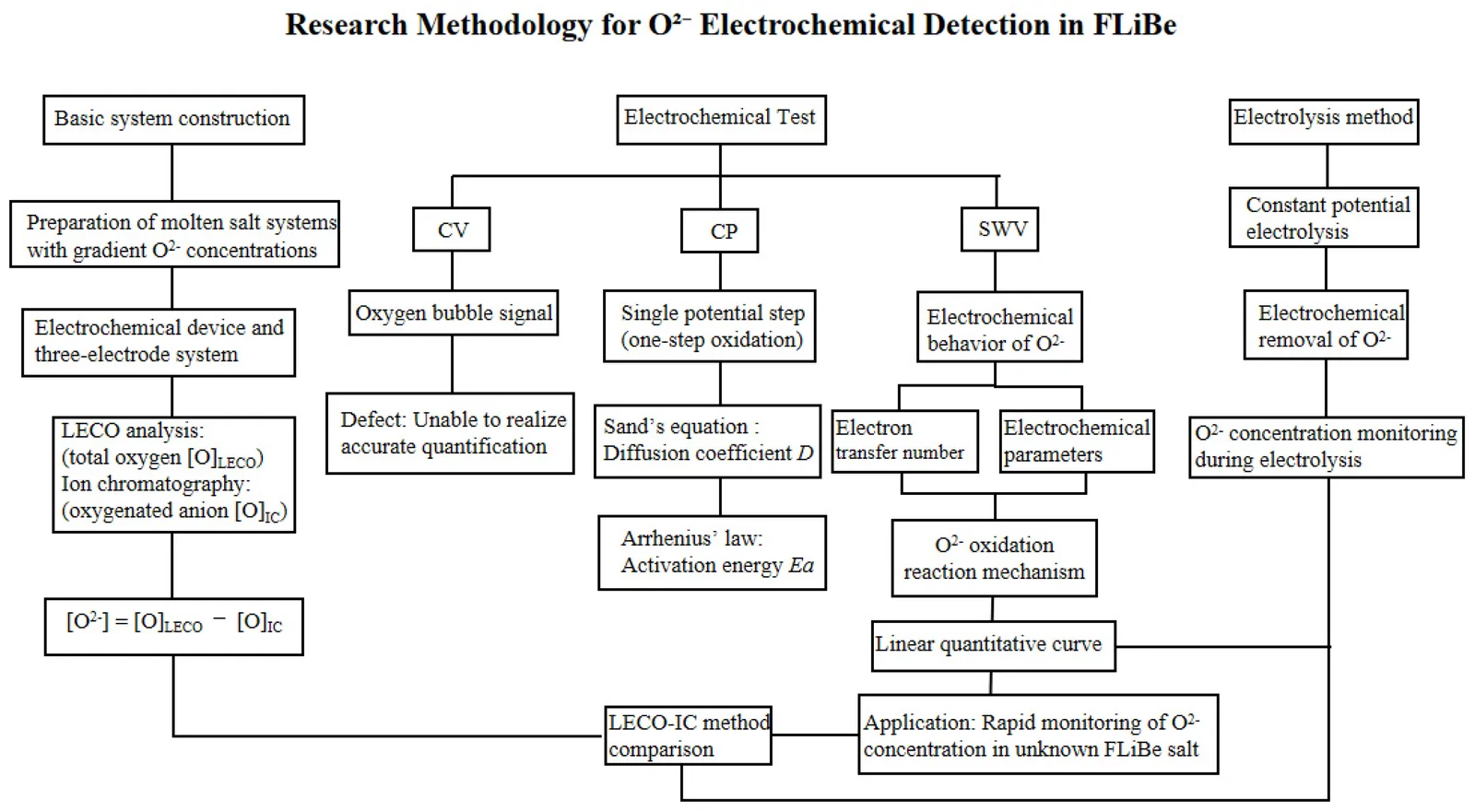
Overall research methodology for this study.

Figure 1 illustrates the overall research methodology adopted in this work.

### 2.2. Preparation of High Purity FLiBe Molten Salt

Due to the inherent hygroscopicity of fluoride salts, the FLiBe preparation process requires strict standards for deoxygenation and purification. The detailed preparation process of high-purity FLiBe molten salt used in this work is described as follows.

The raw materials, LiF (99.9%; Sigma Aldrich; Burlington, MA, USA) and BeF_2_ powders (99.9%; Alfa Aesar; Ward Hill, MA, USA), were placed in a high-purity nickel crucible and vacuum-dried at 300–400 °C to remove trace adsorbed free water, thereby eliminating oxide impurities introduced during the subsequent melting process. Subsequently, the pretreated LiF and BeF_2_ were accurately weighed at a molar ratio of 66.7:33.3 and completely melted at 650 °C. During the melting process, high-purity Ar gas (99.999%; Shanghai Louyang Gas Filling Co., Ltd., Shanghai, China) and H_2_/HF mixed gas (10:1 mol; Shanghai Louyang Gas Filling Co., Ltd., Shanghai, China) with constant pressure and flow rate were continuously purged into the molten salt to further eliminate residual water and oxide impurities. After purification was complete, the HF gas supply was terminated first, and the molten salt was continuously purged with ultra-pure H_2_ for 2–4 h to remove metallic impurity ions and trace dissolved HF in the molten salt. Following H_2_ purging, dry Ar gas was adopted to further remove the residual HF and H_2_ from the molten salt. After the preparation procedure, the liquid FLiBe molten salt was poured into a high-purity nickel crucible and cooled naturally to room temperature for use. All operations were carried out in a glove box protected by argon gas (99.999%).

### 2.3. Preparation of Molten Salts with Different O^2−^ Concentrations

Li_2_O (99.99%; Sigma Aldrich; Burlington, MA, USA) was introduced into the FLiBe molten salt system as the source of O^2−^. A series of FLiBe molten salts with different O^2−^ concentrations was prepared by adjusting the amount of Li_2_O. Different amounts of Li_2_O were added to 40 g of FLiBe molten salt, and the incremental O^2−^ concentration was calculated according to Equation (4).

(4) $${\left[O^{2-}\right]}_{\mathrm{added}}=\frac{1000}{30}\;\times \;\frac{m_{{\mathrm{Li}}_{2}O}}{m_{\mathrm{FLiBe}}}\;(\mathrm{mol}/\mathrm{kg})$$

where [O^2−^]_added_ is the incremental O^2−^ concentration calculated from the added mass of Li_2_O; $m_{{\mathrm{Li}}_{2}O}$ represents the added mass of Li_2_O; $m_{FLiBe}$ is the total mass of FLiBe molten salt, which was fixed at 40 g throughout the experiments.

Table 1 summarizes the addition amounts of Li_2_O in FLiBe molten salt. All subsequent electrochemical measurements were performed in the FLiBe–O^2−^ systems listed in Table 1.

### 2.4. Electrochemical Setup and Measurements

#### 2.4.1. Electrochemical Setup

**Figure 2:**
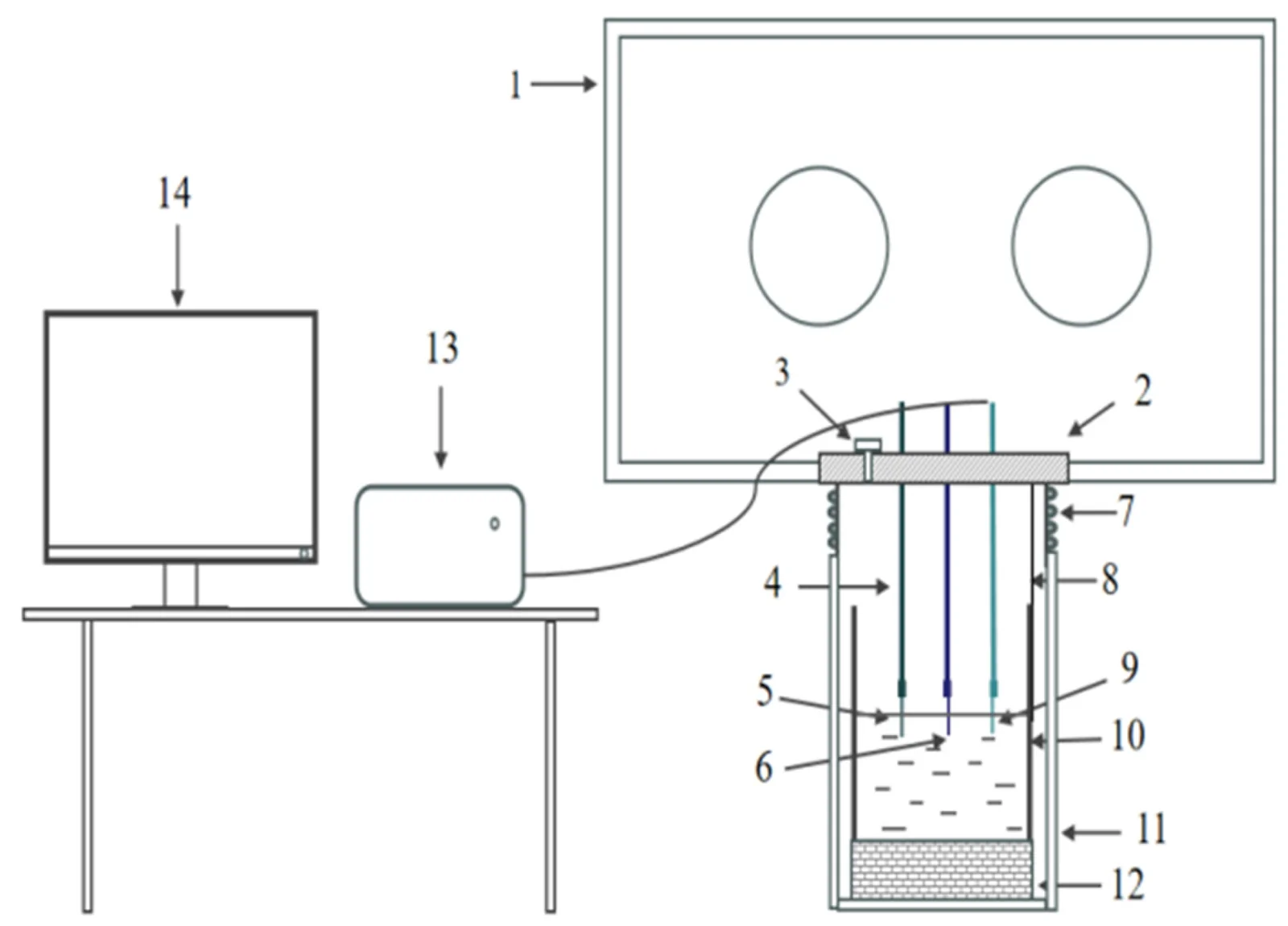
Schematic diagram of the molten salt electrochemical device. (1) Glove box, (2) reactor thermal insulation layer, (3) sampling channel, (4) alumina insulating electrode sleeve, (5) working electrode, (6) reference electrode, (7) water cooling coil let, (8) thermocouple, (9) auxiliary electrode, (10) vitreous carbon crucible with molten salt, (11) electric furnace, (12) alumina insulating sheet, (13) electrochemical workstation, (14) computer and data analysis software (CHI660F).

All the electrochemical experiments were performed in a glove box equipped with an electric furnace and filled with a high-purity Ar atmosphere (99.999% purity). The water and oxygen contents in the glove box were strictly controlled below 0.5 ppm. The schematic diagram of the electrochemical setup used in this study is shown in Figure 2.

The prepared high-purity FLiBe salt (40 g) was accurately weighed and loaded into a vitreous carbon crucible (*Φ* = 50 mm × *H* 40 mm). Then, the crucible loaded with solid eutectic salt was placed and fixed in a stainless steel protective sleeve, which was further transferred inside the furnace. An alumina sheet was laid at the bottom of the sleeve for insulation. The FLiBe salt was heated by a programmed temperature control until it reached 600 °C, and then kept at this temperature to ensure that the salt was fully melted and remained in a homogeneous molten state. The actual temperature of the system was monitored in real time by a nickel–chromium thermocouple closely attached to the outer wall of the vitreous carbon crucible.

#### 2.4.2. Electrodes

**Figure 3:**
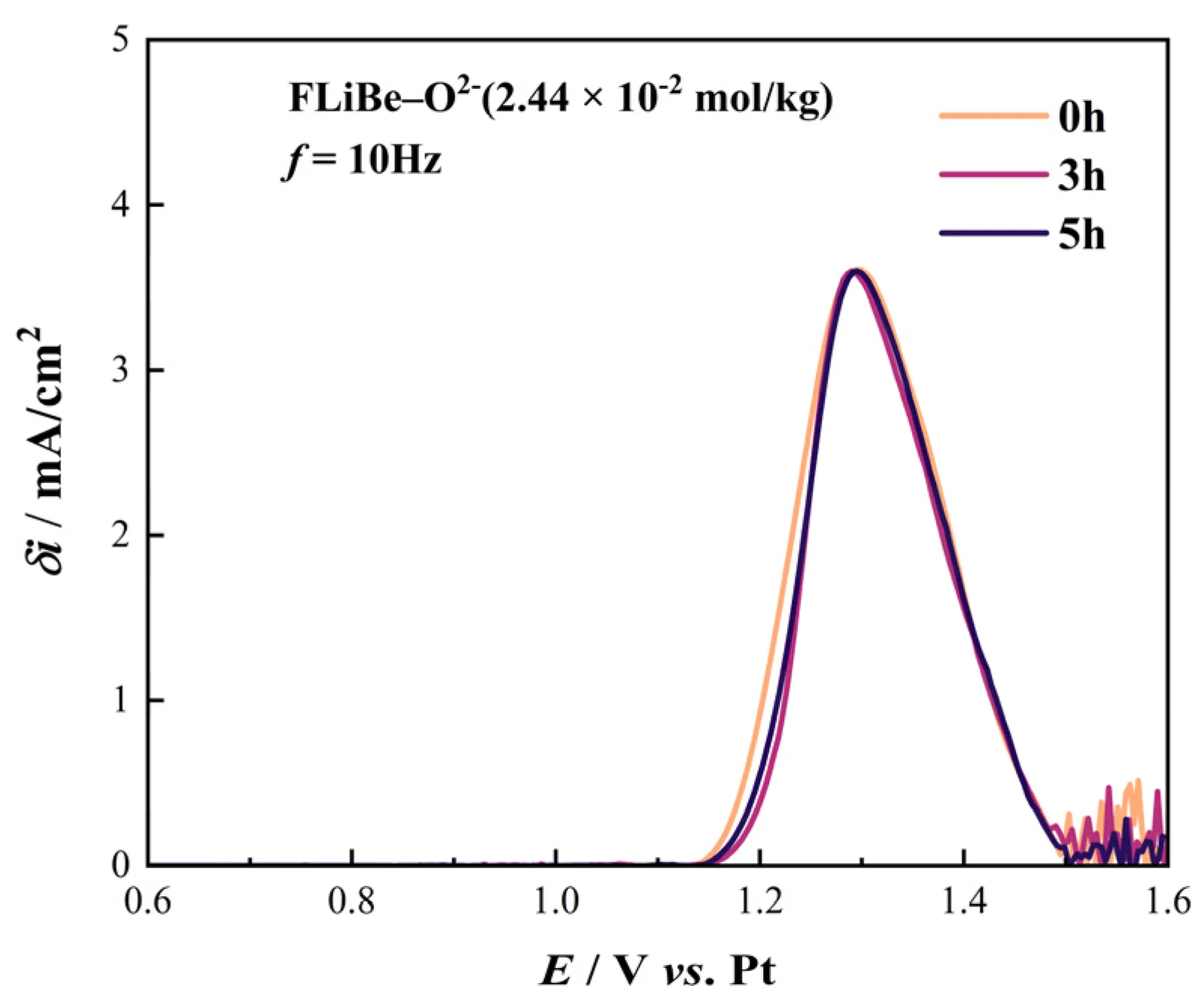
SWV curves of the O^2−^ oxidation in FLiBe–O^2−^ (2.44 × 10^−2^ mol/kg) molten salt at different durations (0 h, 3 h, 5 h). Scan frequency: 10 Hz; pulse height: 25 mV; step potential: 4 mV; working electrode: Au; reference electrode: Pt; auxiliary electrode: graphite.

After the molten salt had fully melted and the system had reached a stable state, a three-electrode system was employed to investigate the electrochemical behavior and electrolytic removal of O^2−^. A gold wire (*Φ* = 1 mm, 99.99%; Sinopharm Chemical Reagent Co., Ltd., Shanghai, China) was used as the working electrode. The Au electrode exhibits excellent chemical inertness in the high-temperature FLiBe molten salt. Since the oxidation potential of O^2−^ is lower than that of gold, and the oxidation product (O_2_) does not react with gold at the experimental temperature, the Au electrode is suitable for the electrochemical testing and electrolysis study of O^2−^ in molten salt systems [28,29]. A graphite rod (*Φ* = 6 mm; Sinopharm Chemical Reagent Co., Ltd., Shanghai, China) was selected as the auxiliary electrode. The large immersion contact area of the graphite auxiliary electrode ensured stable current output. A platinum wire (*Φ* = 1 mm, 99.99%; Sinopharm Chemical Reagent Co., Ltd., Shanghai, China) was adopted to construct a Pt/PtO*_x_*/O^2−^ quasi-reference electrode system [32,33], with a stable potential when the O^2−^ concentration is constant. As shown in Figure 3, under the same O^2−^ concentration condition, the O^2−^ oxidation peak potential measured using the Pt quasi-reference electrode showed no drift within 5 h, indicating the feasibility of using Pt as a reference electrode in this study. After completion of the experiment, the surface area of the working electrode was determined by measuring the immersion depth of the electrode in the molten salt.

#### 2.4.3. Electrochemical Methods

CV, SWV, and CP were utilized to systematically study the electrochemical behavior of O^2−^ in the FLiBe molten salt. Particularly, SWV was employed to establish an electrochemical quantitative analysis method for O^2−^. The oxide removal from the FLiBe–O^2−^ system was carried out by using the potentiostatic electrolysis method. All the electrochemical measurements were performed on a computer-controlled electrochemical workstation (Type CHI660F, Shanghai Chenhua Instruments Co., Ltd., Shanghai, China). The obtained electrochemical experimental data were processed and analyzed using the professional supporting software (CHI660F) matched with the electrochemical workstation.

### 2.5. Chemical Analysis Techniques

#### 2.5.1. Determination of Total Oxide Content in Molten Salt via LECO Oxide Analyzer

**Figure 4:**
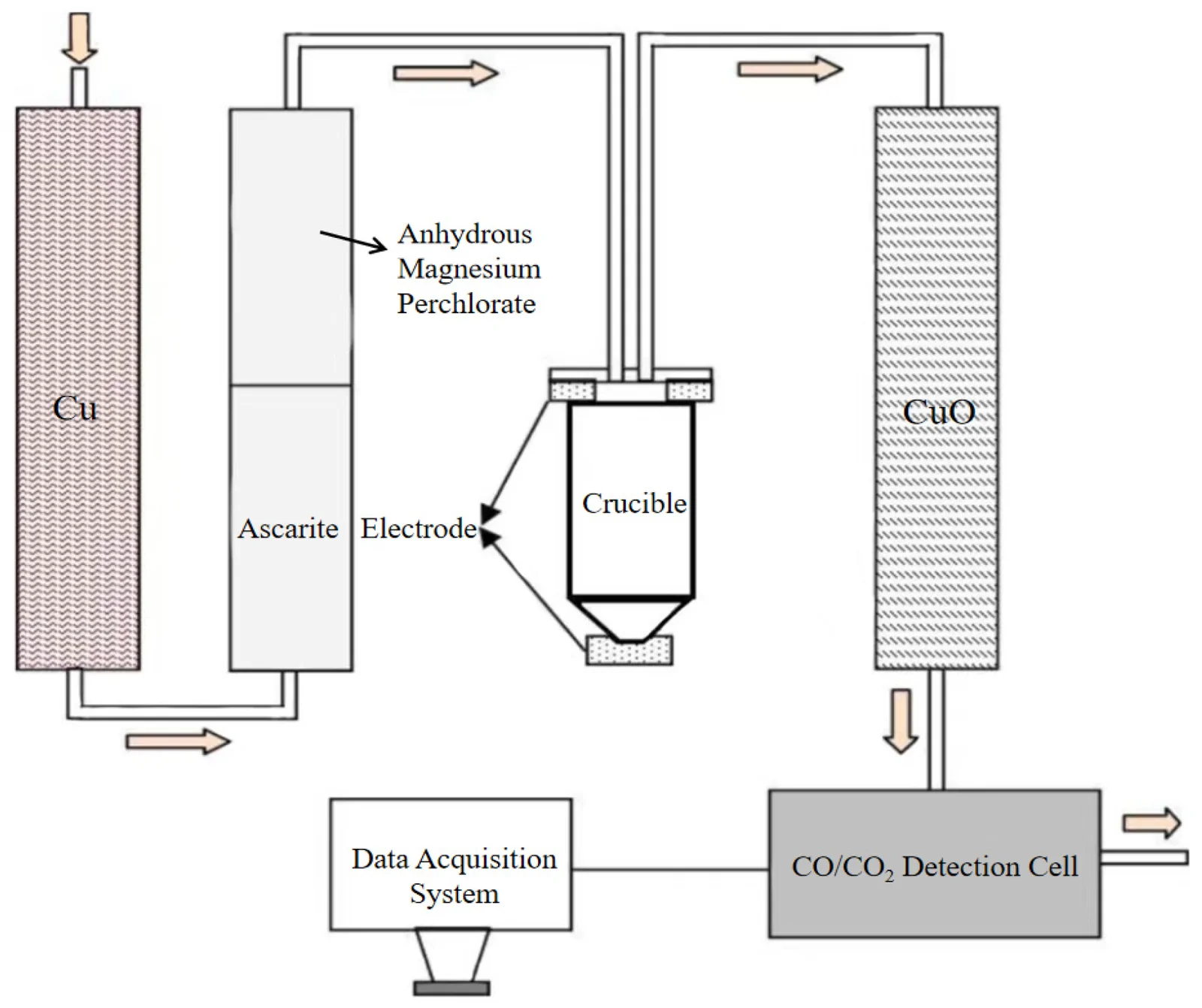
Schematic diagram of the LECO oxide analyzer.

The total oxide content of the molten fluoride salt was determined using an LECO oxide analyzer (Type RO600, LECO Co., Ltd., St. Joseph, MI, USA), whose structure and testing process schematic diagram are shown in Figure 4. This instrument is based on the inert gas fusion-carbon thermal reduction principle, and the obtained results represent the total oxide content of molten salt samples.

For LECO testing, approximately 50 mg of powdered molten salt sample was first wrapped with a dedicated tin capsule in a glove box. Then, the encapsulated sample was placed into a special graphite crucible in the instrument and rapidly heated to 2700 °C by current excitation. At this high temperature, the oxide in the molten salt sample would be released and react with carbon in the graphite crucible to generate CO and CO_2_ gases. The total oxide content was quantitatively calculated according to the infrared detection signals of the generated gases. Prior to testing, the carrier gas was deoxygenated by high-temperature copper wire and further purified by asbestos and anhydrous magnesium perchlorate to remove residual water and CO_2_, eliminating external oxygen interference. To reduce systematic errors and improve testing accuracy, samples were collected from five different positions of the molten salt for parallel tests, and the average value was adopted as the final result.

**Figure 5:**
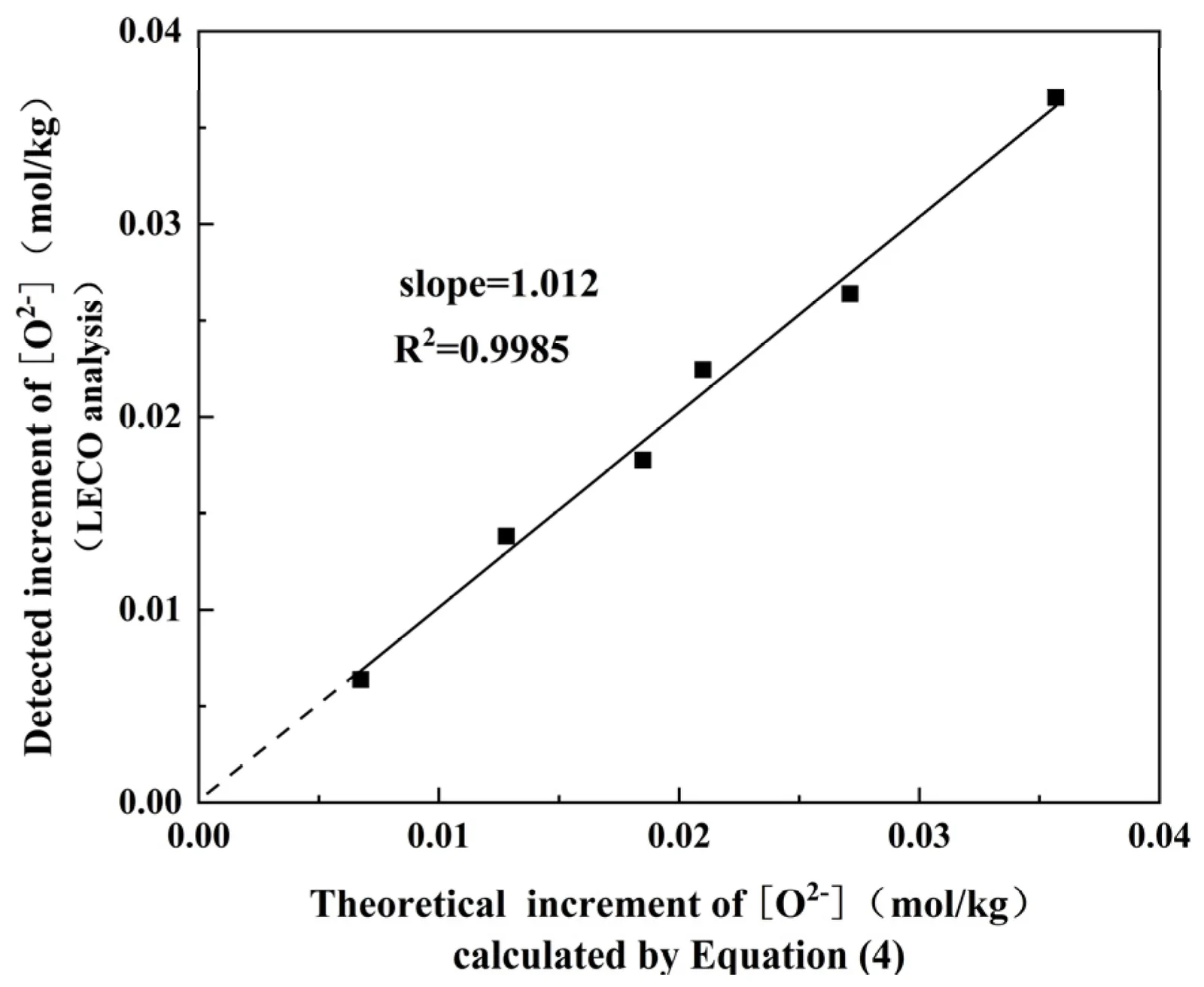
Linear relationship between theoretical increments of O^2−^ concentration (calculated by Li_2_O addition mass) and experimental increments (measured by LECO) in FLiBe molten salt.

In order to verify the accuracy of the LECO oxide analyzer, after addition of different amounts of Li_2_O into the FLiBe melts, LECO was used to detect the increments of O^2−^, which should be consistent with the theoretical weighing values. Figure 5 shows the relationship between the theoretical O^2−^ increments vs. detected O^2−^ increments. The slope is approximately equal to 1, which indicates the LECO is reliable for detecting the oxide content in FLiBe salt. Meanwhile, this result also proved that the added Li_2_O can be completely dissolved in the FLiBe molten salt and fully release O^2−^ ions into the melt.

#### 2.5.2. Determination of Oxygenated Anions Concentration in Molten Salt via Ion Chromatography

**Figure 6:**
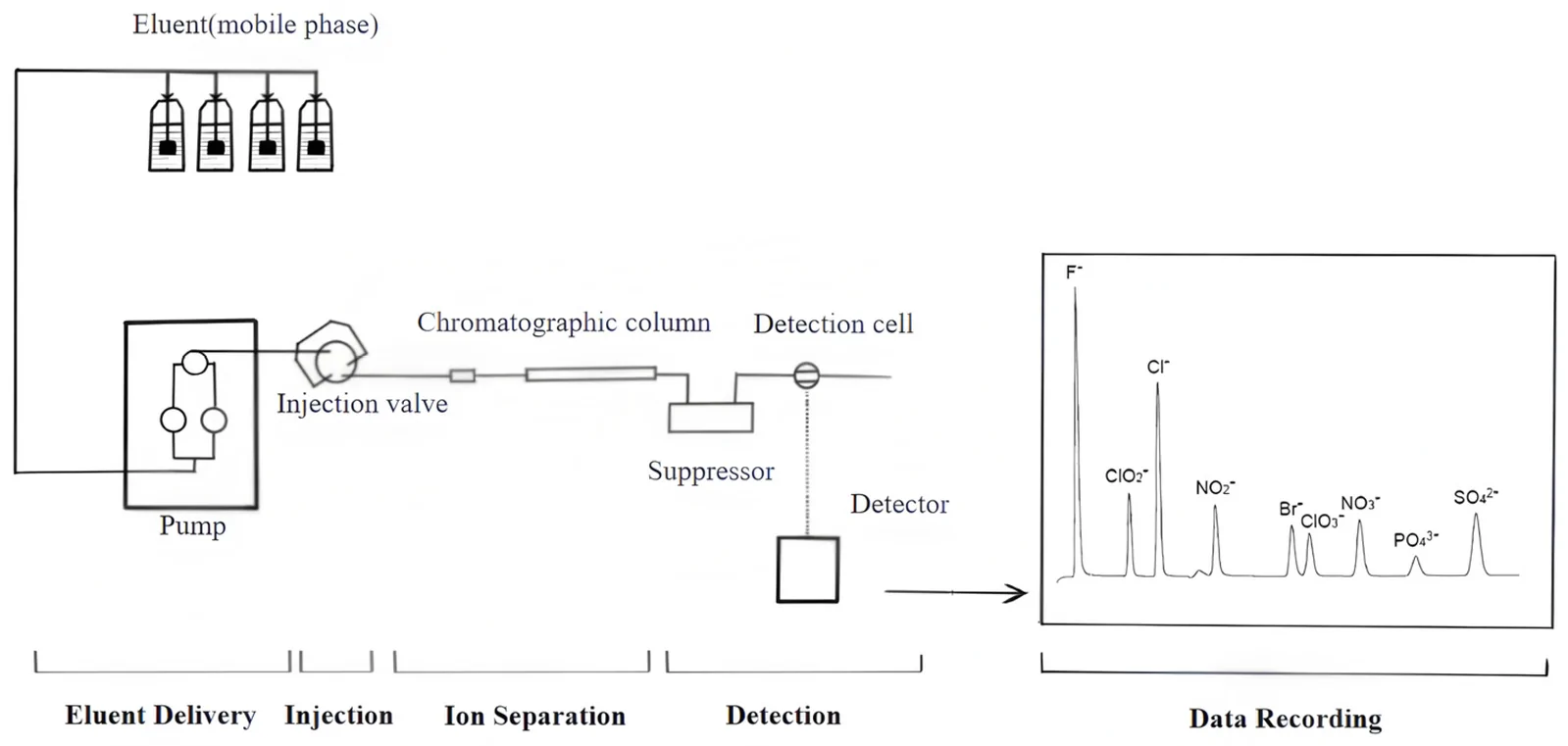
Schematic diagram of the ion chromatography.

An ion chromatograph (ICS-2100, Dionex, Thermo Scientific, Waltham, MA, USA) was employed to determine the contents of oxygenated anions (e.g., NO_3_^−^, PO_4_^3−^, SO_4_^2−^) in molten salt samples, and its schematic diagram is shown in Figure 6. Considering the strong matrix interference caused by high-concentration fluoride ions in FLiBe molten salt, standardized pretreatment and column-switching separation procedures were implemented to guarantee accurate detection of trace anionic impurities. The detailed testing procedures are presented as follows. The detailed testing procedures are presented as follows.

(1) Sample preparation: All sampling vessels were pre-cleaned with ultra-pure water and thoroughly dried to prevent external anion contamination. Approximately 0.30–0.50 g of finely ground molten salt powder was accurately weighed and dissolved in ultra-pure water. The solution was diluted to a fixed volume of 25 mL, shaken evenly, and ultrasonically extracted for 40 min to fully release water-soluble oxygenated anions from the molten salt matrix. After standing to settle fine insoluble particles, the clear supernatant was collected for subsequent chromatographic testing.

(2) Pre-separation treatment: High-content fluoride ions in FLiBe molten salt can interfere with target anion separation. To eliminate such matrix interference, a valve-switching pre-separation strategy was adopted. The prepared sample solution was injected into a sulfonic acid ion-exclusion column. By precise control of switching time and flow rate, the majority of fluoride ions were diverted and discharged as waste, while trace target anions were retained and enriched in the concentration column for subsequent high-sensitivity detection.

(3) Separation and detection: The enriched anions on the concentration column were eluted with matched eluent and then delivered to an ion exchange chromatographic column. Target anions were separated based on their distinct retention characteristics and finally detected by a suppressor conductivity detector.

(4) Result analysis: Under stable chromatographic conditions, each anion exhibits a specific retention time for qualitative identification. Within the calibrated linear range, the anion peak area is linearly correlated with its concentration. Based on the pre-established standard curve, the peak areas of sample chromatograms were analyzed via professional software (ICS-2100) to calculate the concentration of each oxygenated anion, and the impurity content in the original molten salt sample was finally converted.

## 3. Results and Discussion

### 3.1. Oxide Analysis of Molten Salt

Oxygen element primarily exists in fluoride molten salts in the forms of free O^2−^ and various oxygenated anions (e.g., NO_3_^−^, PO_4_^3−^, SO_4_^2−^).

The total oxide content in the molten salt was measured using a LECO oxide analyzer, denoted as [O]_LECO_. The oxygenated anion content was determined via ion chromatography (IC). The oxide concentration derived from all the oxygenated anions was calculated according to the oxide proportion in each oxygenated anion, defined as [O]_IC_. Consequently, the actual content of free O^2−^ ions in the molten salt, expressed as [O^2−^], can be obtained by subtracting the oxide in oxygenated anions ([O]_IC_) from the total oxide content ([O]_LECO_), as shown in Equation (5).

(5) $$\left[O^{2-}\right]={\left[O\right]}_{\mathrm{LECO}}-{\left[O\right]}_{\mathrm{IC}}$$

The oxide analysis results of FLiBe molten salt are presented in Table 2. The total oxide concentration of the molten salt is 1.42 × 10^−2^ mol/kg; three types of oxygenated anions, namely NO_3_^−^, SO_4_^2−^, and PO_4_^3−^, were detected, with concentrations of 3.06 × 10^−4^, 1.25 × 10^−4^, and 1.05 × 10^−4^ mol/kg, respectively. After subtracting the total oxide content in these oxygenated anions (1.84 × 10^−3^ mol/kg), the actual concentration of free O^2−^ in the original FLiBe molten salt was determined to be 1.24 × 10^−2^ mol/kg.

### 3.2. Electrochemical Behavior of O^2−^ in FLiBe

#### 3.2.1. Cyclic Voltammetry

**Figure 7:**
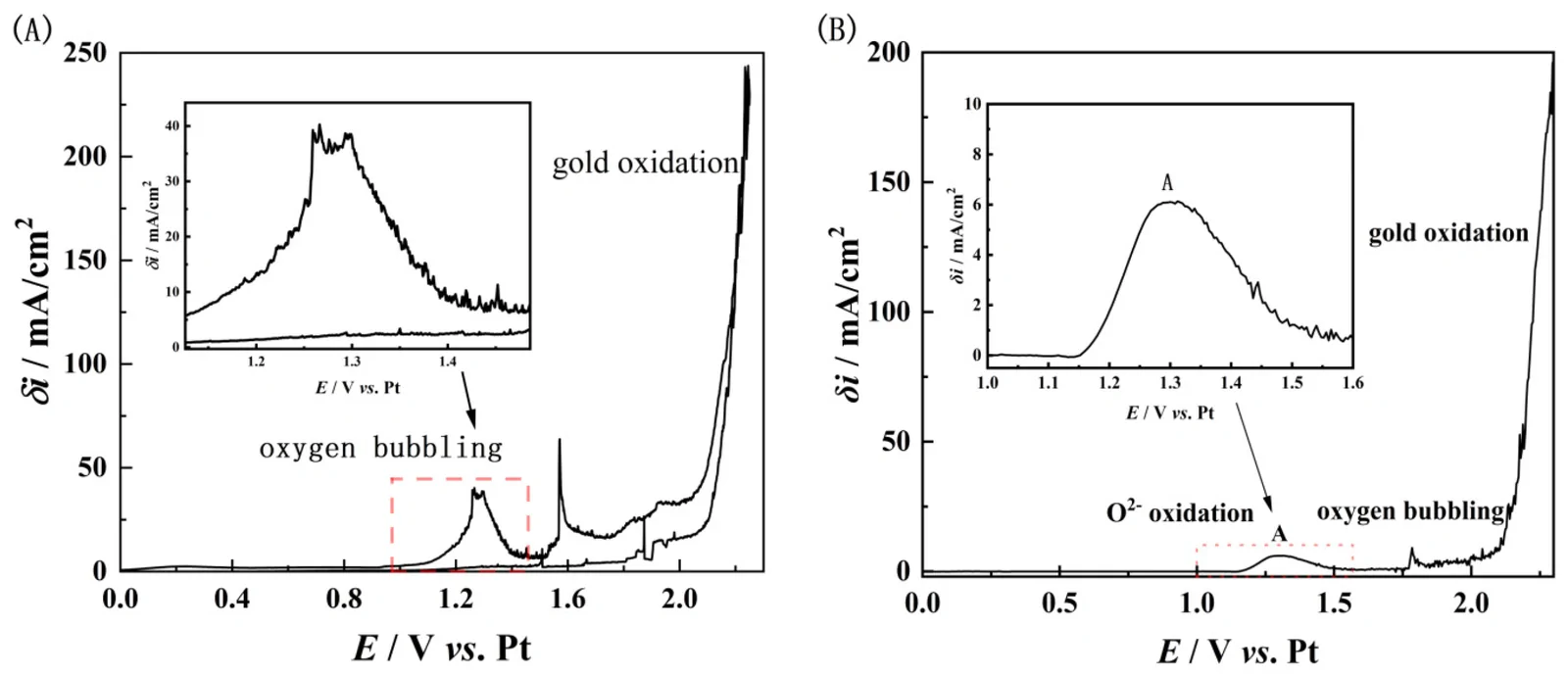
(**A**) Cyclic voltammogram of the FLiBe–O^2−^ (6.18 × 10^−2^ mol/kg) molten salt system at a scan rate of 0.1 V/s and T = 600 °C. Inset: local magnified view of the O^2−^ oxidation signal; working electrode: Au; reference electrode: Pt; auxiliary electrode: graphite. (**B**) Square wave voltammogram of the FLiBe–O^2−^ (2.03 × 10^−2^ mol/kg) molten salt system. *T* = 600 °C. Inset: local magnified view of the O^2−^ oxidation peak; scan frequency: 25 Hz; pulse height: 25 mV; step potential: 4 mV; working electrode: Au; reference electrode: Pt; auxiliary electrode: graphite.

CV was employed to investigate the electrochemical behavior of O^2−^ in FLiBe molten salt, and the obtained cyclic voltammogram is presented in Figure 7A. It can be seen that the CV of the FLiBe–O^2−^ system is somewhat disturbed by gas bubbling. The anodic run of the CV curve shows that the oxidation current begins to increase at approximately 1.1 V vs. Pt. Obvious signal disturbances emerge on the working curve when the potential rises to 1.2 V vs. Pt, which is presumably associated with gas evolution, indicating that O^2−^ is oxidized to O_2_ at this potential. Within the potential range of 1.2–2.0 V vs. Pt, the continuous generation of O_2_ induces intense current disturbances, resulting in baseline drift and peak shape distortion. As shown in the inset of Figure 7A, the position of the oxidation peak is difficult to determine. The interference caused by oxygen bubble evolution severely compromises the accurate calibration of the oxidation peak current with the O^2−^ content. Consequently, CV is not suitable for investigating the electrochemical behavior of O^2−^ in FLiBe molten salt, which greatly limits its application in the electrochemical quantitative analysis of O^2−^.

#### 3.2.2. Square Wave Voltammetry

SWV, as a high-precision electrochemical analysis technique, was employed to investigate the electrochemical behaviors of O^2−^ in FLiBe molten salt. Figure 7B presents the typical square wave voltammogram of the FLiBe–O^2−^ (2.03 × 10^−2^ mol/kg) system measured on a gold electrode at 600 °C. A distinct oxidation peak A appears at approximately 1.3 V vs. Pt. Unlike CV results, this oxidation peak exhibits a well-defined peak with a clear shape and peak position, which is hardly affected by the disturbing effects of oxygen. This phenomenon confirms that SWV is more suitable for electrochemical analysis of O^2−^. Evidently, peak A corresponds to the oxidation of O^2−^. After 2.1 V vs. Pt, the oxidation current significantly increased, which was attributed to the oxidation and dissolution of the Au working electrode into the molten salt. The oxidation of the Au electrode was also observed in CV when the potential exceeded 2.1 V vs. Pt.

**Figure 8:**
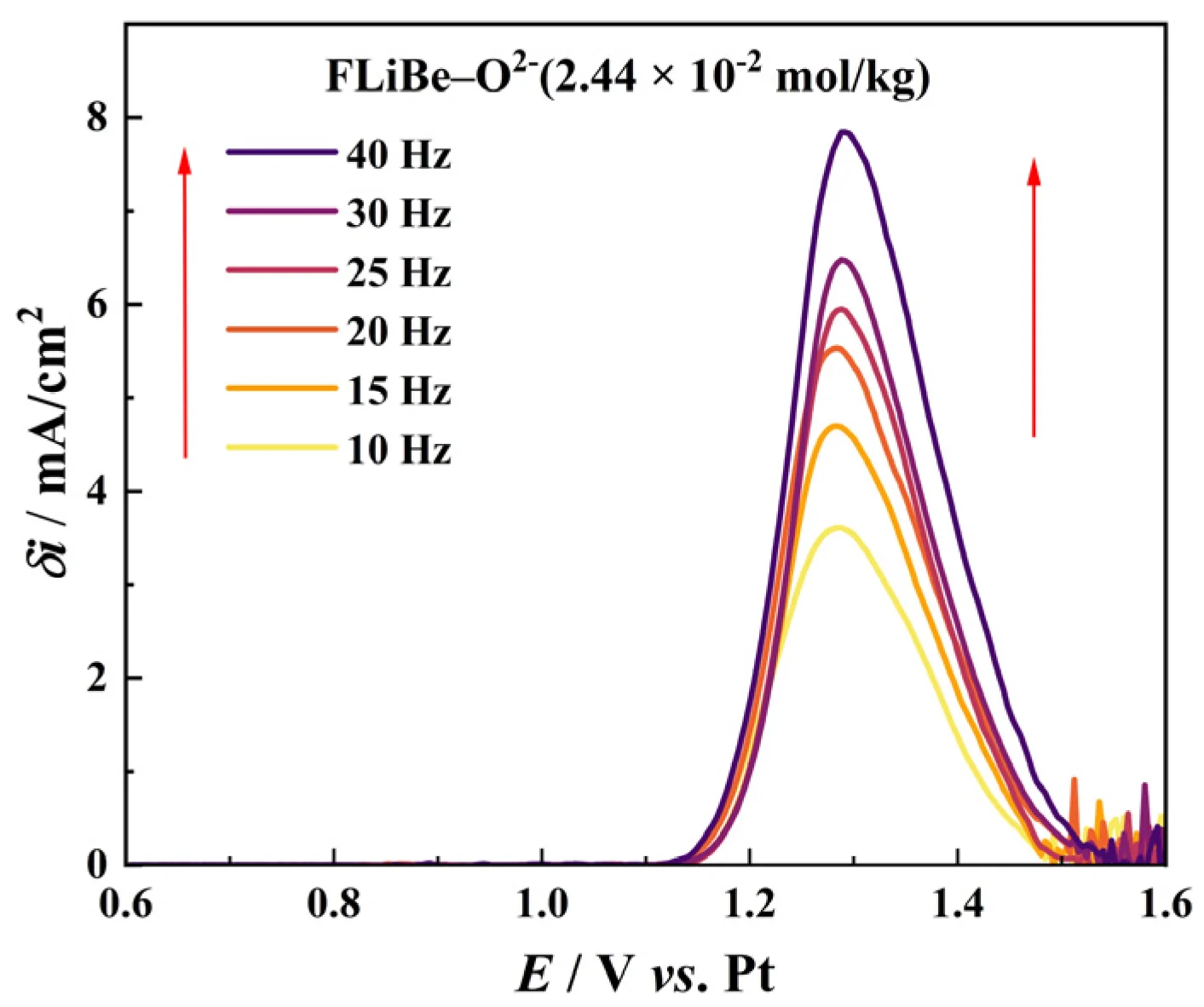
Square wave voltammograms of the FLiBe–O^2−^ (2.44 × 10^−2^ mol/kg) molten salt system at different scan frequencies of 10–40 Hz. *T* = 600 °C; pulse height: 25 mV; step potential: 4 mV; working electrode: Au; reference electrode: Pt; auxiliary electrode: graphite.

**Figure 9:**
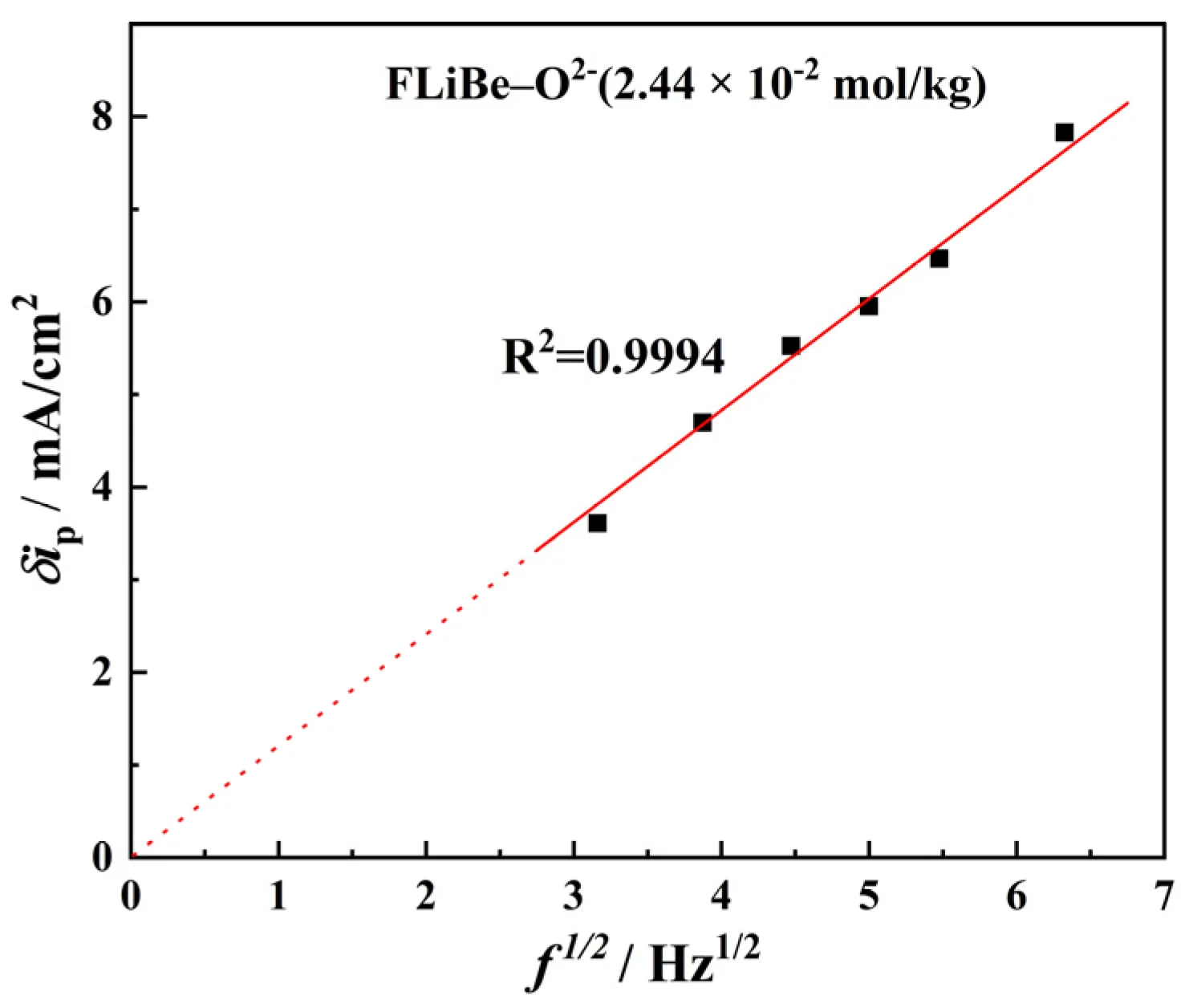
Linear relationship between the peak current density and the square root of the frequency in the FLiBe–O^2−^ (2.44 × 10^−2^ mol/kg) molten salt system. *T* = 600 °C; pulse height: 25 mV; step potential: 4 mV; working electrode: Au; reference electrode: Pt; auxiliary electrode: graphite.

Theoretically, SWV electrochemical analysis is primarily applicable to reversible electrochemical systems, while it can also be extended to systems where the peak current exhibits a linear relationship with the square root of the scanning frequency. In this study, the peak current of O^2−^ gradually increases with increasing signal frequency, as shown in Figure 8. The peak intensity is linear with the square root of the signal frequency in the 10–40 Hz frequency range, and the fitted straight lines pass through the origin, as shown in Figure 9. This result confirms that the oxidation of O^2−^ is controlled by ion diffusion. Therefore, SWV is suitable for further analysis of the FLiBe–O^2−^ electrochemical system.

According to the SWV theory, for diffusion-controlled electrochemical processes, the electron transfer number of the electrode reaction can be determined by measuring the half-peak width (*W*_1/2_), as shown in Equation (6).

(6) $$W_{1/2}=3.52\;\times \;\frac{\mathrm{RT}}{\mathrm{nF}}$$

where *W*_1/2_ is the half-peak width (V); *R* is the ideal gas constant (8.314 J/(mol.K)); *T* represents the absolute temperature (K); *n* is the electron transfer number; *F* is the Faraday constant (96,485 C/mol).

Different from the symmetric peak shape observed in conventional soluble/soluble electrochemical systems, the oxidation peak of O^2−^ is not exactly symmetric, i.e., the increasing part of the current is sharper than the decreasing one, as shown in Figure 8. This phenomenon originates from the nucleation overpotential of oxygen gas formation. The overpotential produced during O_2_ gas evolution delays the current-increase response, rendering a steeper rise in the peak current. That means the decreasing part (the right half) of the peak was not affected by the nucleation overpotential. Thus, the disturbance of the signal due to the nucleation effect can be resolved by taking into account only the decreasing part (right half) of the peak for the measurement of the half peak width (*W*_1/2_). Previous literature reports similar methods for determining *W*_1/2_ in the case of the nucleation effect [28,29]. Thus, based on the obtained *W*_1/2_ and Equation (6), it was found that the number of transferred electrons for O^2−^ oxidation at different frequencies was approximately 2, as shown in Table 3. Accordingly, the electrode reaction mechanism for O^2−^ oxidation is ultimately determined as follows:

(7) $$O^{2-}\;=\;1/2O_{2}(g)\;+\;2e^{-}$$

#### 3.2.3. Chronopotentiometry

**Figure 10:**
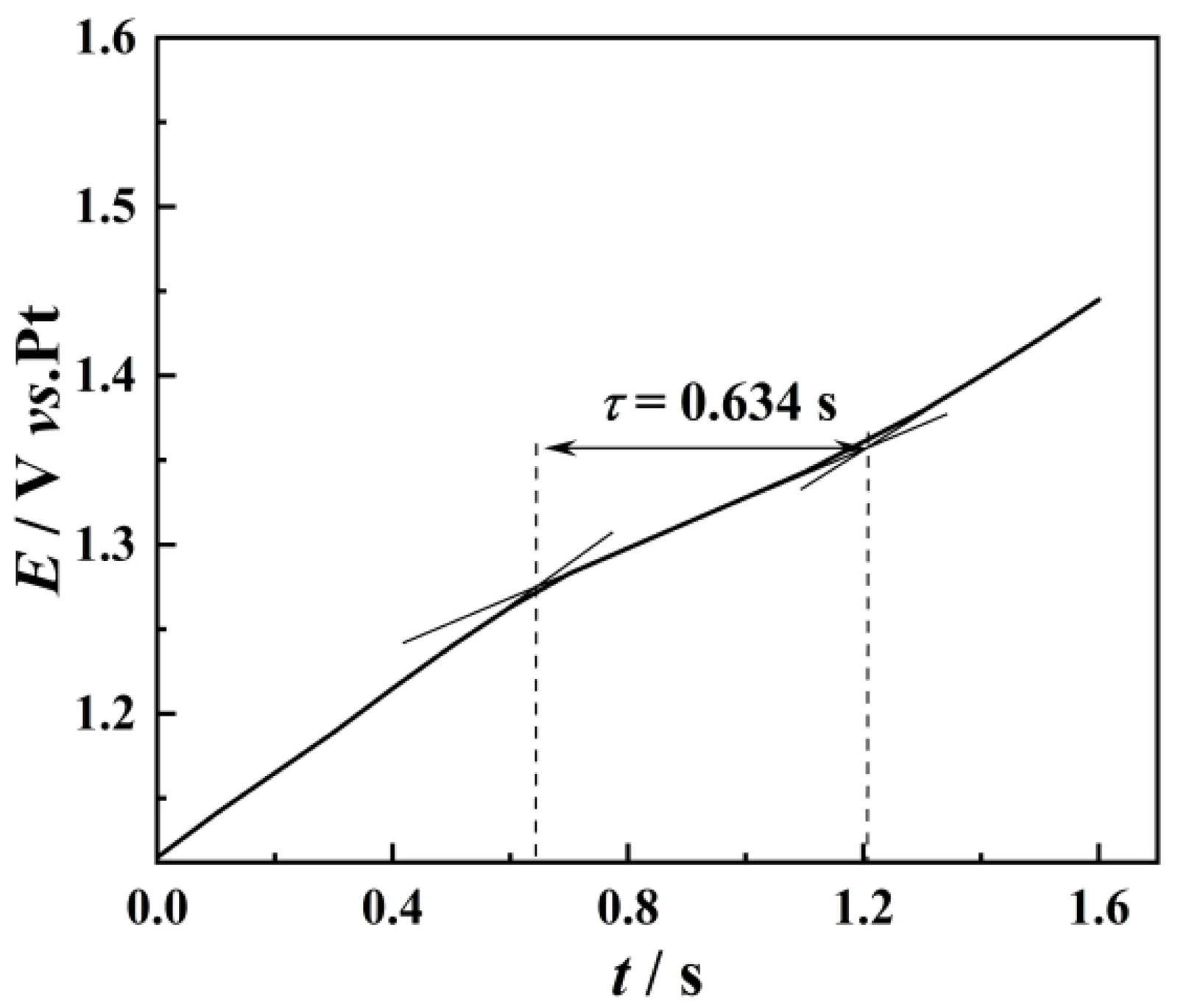
Typical chronopotentiogram of the FLiBe–O^2−^ (3.86 × 10^−2^ mol/kg) molten salt system under an applied current density of 0.89 mA/cm^2^. *T* = 600 °C; working electrode: Au (*S* = 0.121 cm^2^); reference electrode: Pt; auxiliary electrode: graphite. The density of FLiBe melt (ρ_FLiBe_) is approximately 2 g/cm^3^.

**Figure 11:**
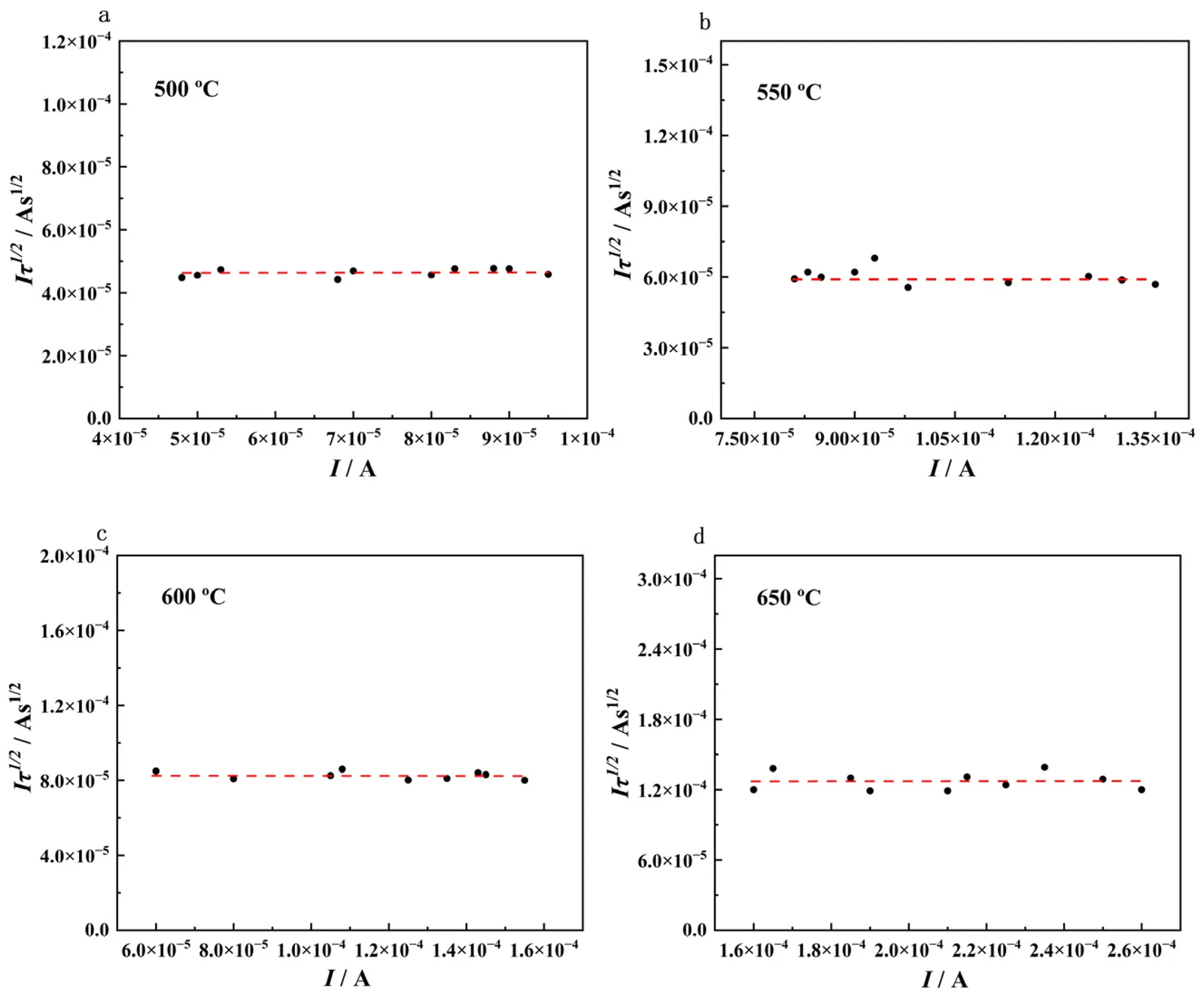
Evolution of *Iτ*^1/2^ vs. the applied current intensity for the FLiBe–O^2−^ (3.86 × 10^−2^ mol/kg) molten salt system at different temperatures. (**a**) 500 °C; (**b**) 550 °C; (**c**) 600 °C; (**d**) 650 °C. Working electrode: Au (S = 0.121 cm^2^); reference electrode: Pt; auxiliary electrode: graphite. The density of FLiBe melt (ρ_FLiBe_) is approximately 2 g/cm^3^.

CP was employed to further investigate the electrochemical behavior and diffusion coefficient of O^2−^ in FLiBe molten salt. The corresponding chronopotentiogram is displayed in Figure 10. For the FLiBe–O^2−^ (3.86 ×10^−2^ mol/kg) system at 600 °C, a potential plateau was observed at approximately 1.3 V vs. Pt under an applied current density of 0.89 mA/cm^2^. The potential of this plateau was consistent with the oxidation peak potential obtained from SWV measurements, and the corresponding transition time (τ) was determined to be 0.634 s. Apparently, this plateau corresponds to the electrochemical oxidation of O^2−^ to O_2_ (Equation (7)). The CP result is in good agreement with that obtained by SWV. Then, CP measurements were performed at a fixed O^2−^ concentration (3.86 × 10^−2^ mol/kg), electrode area (0.121 cm^2^), and temperature (600 °C) by applying different current densities of 0.50, 0.66, 0.87, 0.89, 1.03, 1.12, 1.18, 1.20, and 1.28 mA/cm^2^. The results indicate that τ decreases with increasing current density, and the values of *Iτ*^1/2^ remain nearly constant (Figure 11c). This result is consistent with the Sand law (Equation (8)), further demonstrating that the oxidation of O^2−^ is an ion diffusion-controlled process, which enables the determination of the diffusion coefficient of O^2−^. As shown in Figure 11c, the *Iτ*^1/2^ value remained stable at 8.24 × 10^−5^ As^1/2^ at 600 °C. Accordingly, the diffusion coefficient of O^2−^ in FLiBe molten salt at 600 °C was calculated to be 2.74 × 10^−9^ cm^2^/s based on the Sand law.

(8) $$\frac{I\sqrt{\tau }}{C}=0.5\mathrm{nFS}\sqrt{\pi D}$$

where *S* is the electrode area, in cm^2^; *I* is the applied current, in A; *τ* is the transition time, in s; *C* is the O^2−^ concentration, in mol/cm^3^; *F* is the Faraday constant, valued at 96,485 C/mol; *n* is the number of electrons transferred; *D* is the diffusion coefficient, in cm^2^/s.

CP were performed at different temperatures of 500 °C, 550 °C, 600 °C, and 650 °C. At all tested temperatures, the experimental results show that the transition time (*τ*) decreases with the increase in applied current density (*I*), and the values of *Iτ*^1/2^ remain constant at each temperature. In addition, the constant *Iτ*^1/2^ value increases with rising temperature, as depicted in Figure 11. These results are consistent with the Sand law, i.e., the *Iτ*^1/2^ value increases with temperature (or diffusion coefficient *D*) when the O^2−^ concentration (*C*) and electrode area (*S*) remain constant. Thus, the oxidation of O^2−^ is controlled by ion diffusion over the studied temperature range.

**Figure 12:**
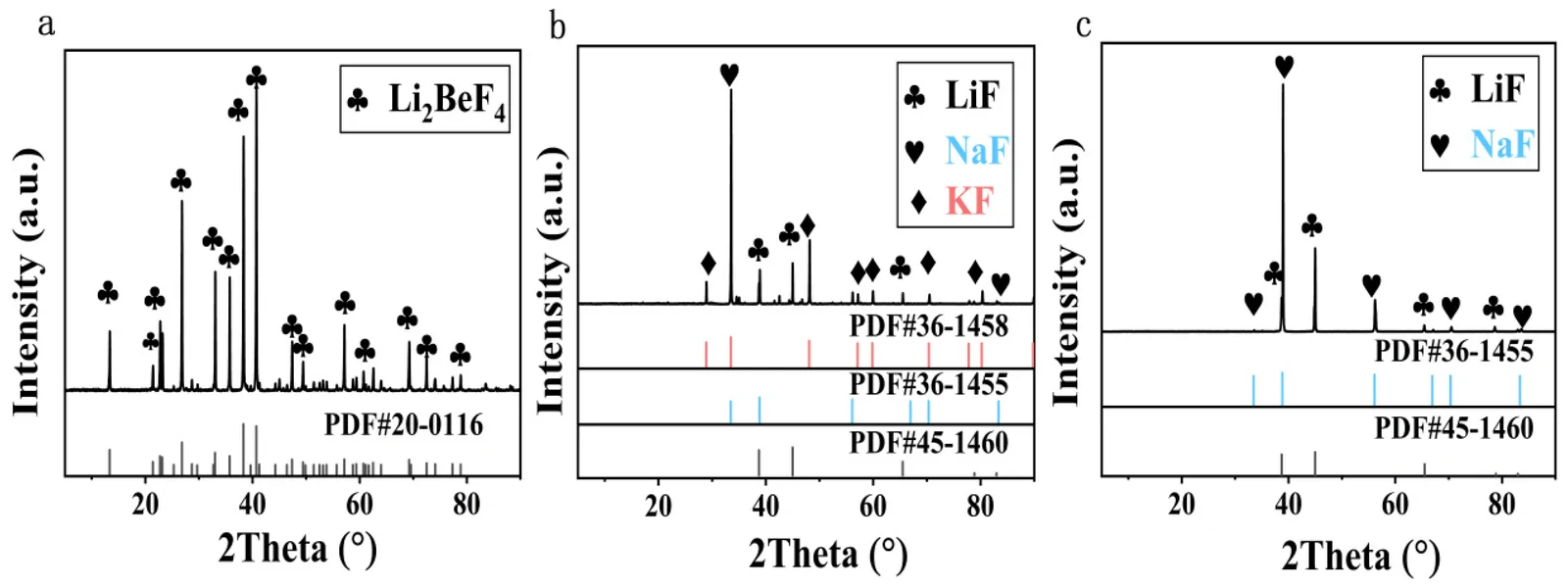
XRD patterns of the (**a**) FLiBe, (**b**) FLiNaK, and (**c**) LiF–NaF (60:40 mol%) molten salts.

Based on the Sand law, the diffusion coefficients (*D*) of O^2−^ in FLiBe melts were obtained at each temperature, and the resulting $D_{O^{2-}}$ increases from 8.55 × 10^−10^ to 6.44 × 10^−9^ cm^2^/s with rising temperature from 500 °C to 650 °C. The $D_{O^{2-}}$ were compared with the reported data in FLiNaK and LiF–NaF systems, as summarized in Table 4. The results indicate that the diffusion coefficient of O^2−^ in FLiBe molten salt is about three orders of magnitude lower than that in FLiNaK and LiF–NaF molten salt systems within the investigated temperature range. The significant difference originates from the unique microstructure and thermophysical properties of FLiBe molten salt: (1) Be^2+^ can coordinate with F^−^ to form [BeF_4_]^2−^ (Figure 12a), which further polymerizes to construct a dense network structure [34]. These molecules have a large volume, thereby hindering the migration of O^2−^ ions. In contrast, FLiNaK and LiF–NaF dissociate completely into free alkali metal ions and fluoride ions at high temperatures without complex formation (Figure 12b,c), resulting in a relatively high O^2−^ diffusion coefficient. (2) Be^2+^ in FLiBe molten salt exhibits favorable coordination ability and can form stable Be^2+^-O^2−^ complex species with O^2−^. The formation of such complexes with relatively large volume and relatively large hydrodynamic radius further reduces the ion migration rate and increases the viscosity of the molten salt. (3) FLiBe molten salt possesses inherently higher viscosity compared with FLiNaK and LiF–NaF systems, which is unfavorable for the mass transfer and migration of O^2−^. The above factors substantially reduce the diffusion rate of O^2−^ in FLiBe molten salt. Therefore, it is reasonable that the diffusion coefficient of O^2−^ in FLiBe (order of magnitude of ~10^−9^) is much lower than that in FLiNaK and LiF–NaF.

**Figure 13:**
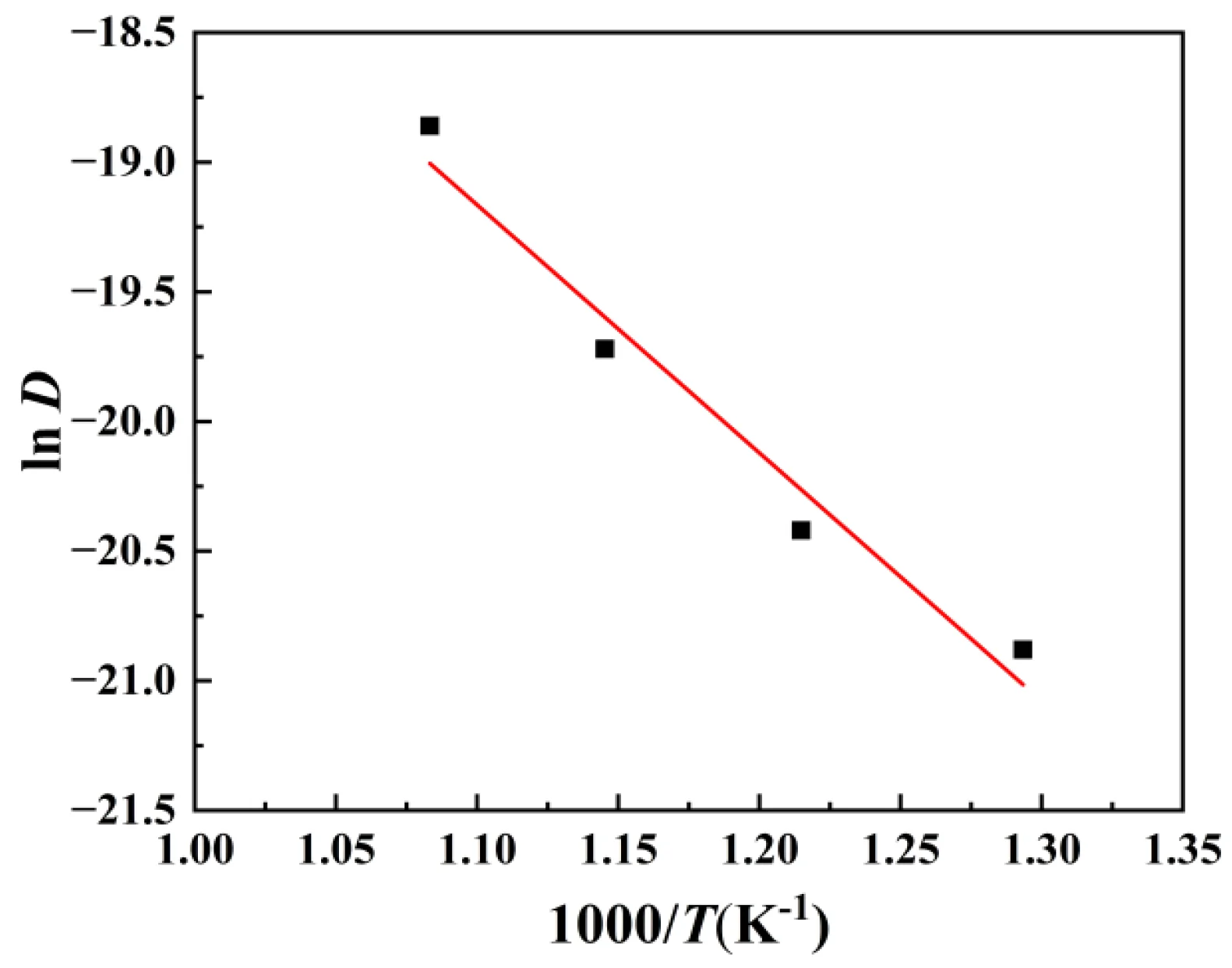
Linear relationship between ln*D* and 1/*T*.

The diffusion coefficient of O^2−^ in FLiBe molten salt increases with the elevation of temperature, and the evolution of *D* with temperature obeys Arrhenius’ law (Equation (9)). By fitting the experimental data based on the Arrhenius formula, as shown in Equation (10) and Figure 13, the activation energy (*E*a) for O^2−^ diffusion in FLiBe molten salt was determined to be 79.5 kJ/mol.

(9) $$D=D^{0}\exp\left(-\frac{Ea}{\mathrm{RT}}\right)$$

(10) $$\ln D_{\left({\mathrm{cm}}^{2}s^{-1}\right)}=-8.64676\;-\frac{9561.997}{T_{\left(K\right)}}$$

### 3.3. Establishment of Quantitative Relationship Between Peak Current Density and O^2−^ Concentration

**Figure 14:**
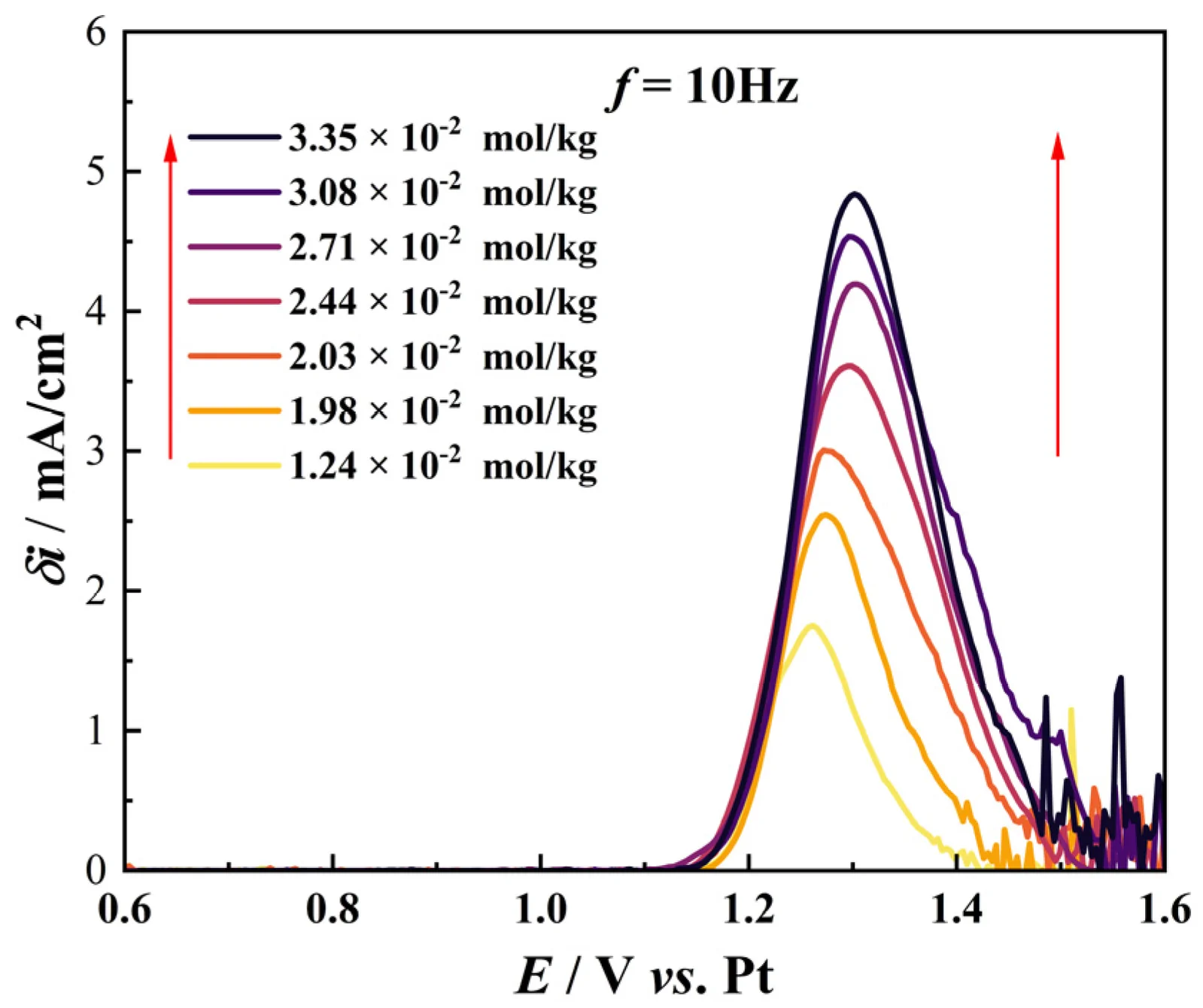
Square wave voltammograms of the FLiBe molten salt containing different O^2−^ concentrations. *T* = 600 °C; scan frequency: 10 Hz; pulse height: 25 mV; step potential: 4 mV; working electrode: Au; reference electrode: Pt; auxiliary electrode: graphite.

**Figure 15:**
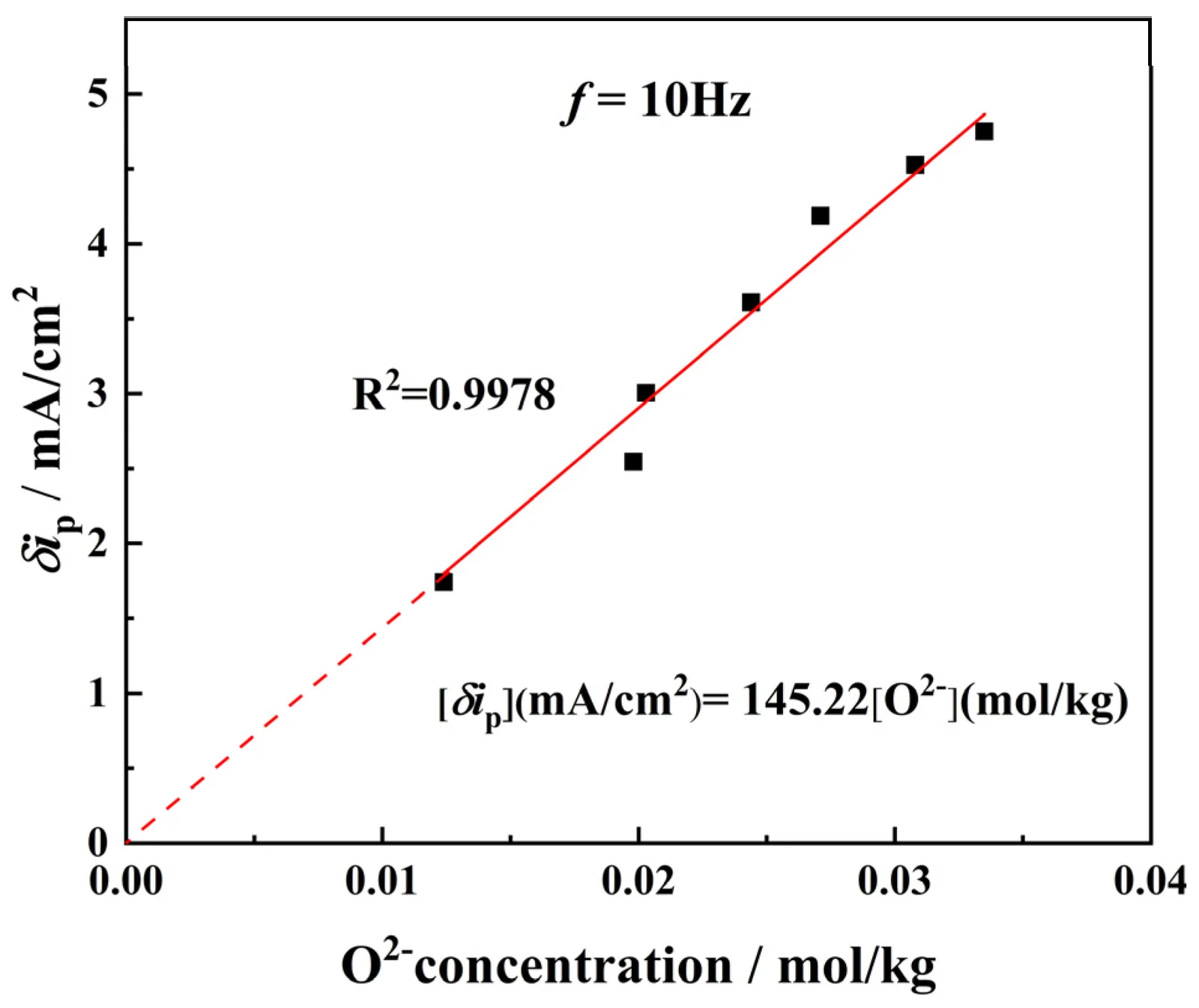
Linear relationship between O^2−^ concentration and peak current density obtained by square wave voltammetry in the FLiBe molten salt containing different O^2−^ concentrations. *T* = 600 °C; scan frequency: 10 Hz; pulse height: 25 mV; step potential: 4 mV; working electrode: Au; reference electrode: Pt; auxiliary electrode: graphite.

Theoretically, for diffusion-controlled electrode reactions, the peak current density should be linear with the reactant concentration. SWVs were performed at 600 °C with a scanning frequency of 10 Hz for FLiBe molten salt containing different O^2−^ concentrations, and the corresponding square wave voltammograms are presented in Figure 14. As the O^2−^ concentration increased from 1.24 × 10^−2^ mol/kg to 3.35 ×10^−2^ mol/kg, the oxidation peak current density of O^2−^ accordingly increased from 1.740 mA/cm^2^ to 4.750 mA/cm^2^, as shown in Table 5. Then, the peak current density vs. O^2−^ concentration is fitted, and a straight line is obtained, as shown in Figure 15. This linear relationship can be described by Equation (11). The slope equals 145.22, and the line passes through the origin of the coordinates, indicating that no O^2−^ oxidation peak current can be detected with no O^2−^ contained in the FLiBe melts. Equation (11) provides a quantitative relationship between the peak current and the O^2−^ concentration. Using this equation, the O^2−^ content in an unknown FLiBe melt can be quickly obtained based on the measured peak current.

(11) $$\delta i_{p}(\mathrm{mA}/cm^{2})\;=\;145.22\;[O^{2-}](\mathrm{mol}/\mathrm{kg})$$

However, the peak potential of O^2−^ positively shifts from 1.262 V to 1.302 V vs. Pt as the O^2−^ concentration increases from 1.24 × 10^−2^ to 3.35 × 10^−2^ mol/kg, which may result from the following two reasons. One is the potential variation in the Pt/PtO_x_/O^2−^ reference electrode, and the other is due to the increase in the activity of O^2−^ according to the Nernst equation (Equation (12)) expressed as follows:

(12) $$E_{O^{2-}/O_{2}}\;=\;E^{0}+\frac{RT}{2F}ln\frac{{\left(P_{O_{2}}/P^{\theta }\right)}^{1/2}}{\alpha_{O^{2-}}}$$

where $E^{0}$ is the standard potential of O^2−^/O_2_ and $\alpha_{O^{2-}}$ is the O^2−^ activity that can be denoted as $\alpha_{O^{2-}}=C_{O^{2-}}\times \gamma$, in which $C_{O^{2-}}$ is the concentration of O^2−^ and γ is its activity coefficient. $P_{O_{2}}$ is the partial pressure of O_2_ and $P^{\theta }$ is the standard atmospheric pressure. The potential of O^2−^/O_2_ with $C_{O^{2-}}$ = 1.24 × 10^−2^ mol/kg is defined as *E*_1_, and the activity coefficient of O^2−^ (γ) is assumed to be 1. Thus, Δ*E* is the difference between *E*_1_ and the potential obtained at $C_{O^{2-}}$ of 1.98 × 10^−2^, 2.03 × 10^−2^, 2.44 × 10^−2^, 2.71 × 10^−2^, 3.08 × 10^−2^ and 3.35 × 10^−2^ mol/kg can be expressed as Equation (13):

(13) $$∆E\;=\;\frac{RT}{2F}ln\frac{C_{1}}{C}$$

where *C*_1_ is 1.24 × 10^−2^ mol/kg, and *C* is the O^2−^ concentration at the other six concentrations, as listed in Table 6. The potential differences in ΔE obtained by using SWV and the Nernst equation are also listed in Table 6. Therefore, the potential variation caused by the Pt/PtO_x_/O^2−^ reference electrode can be quantified by the difference value in the SWV measurements and the Nernst equation calculations.

### 3.4. O^2−^ Content Determination by SWV

**Figure 16:**
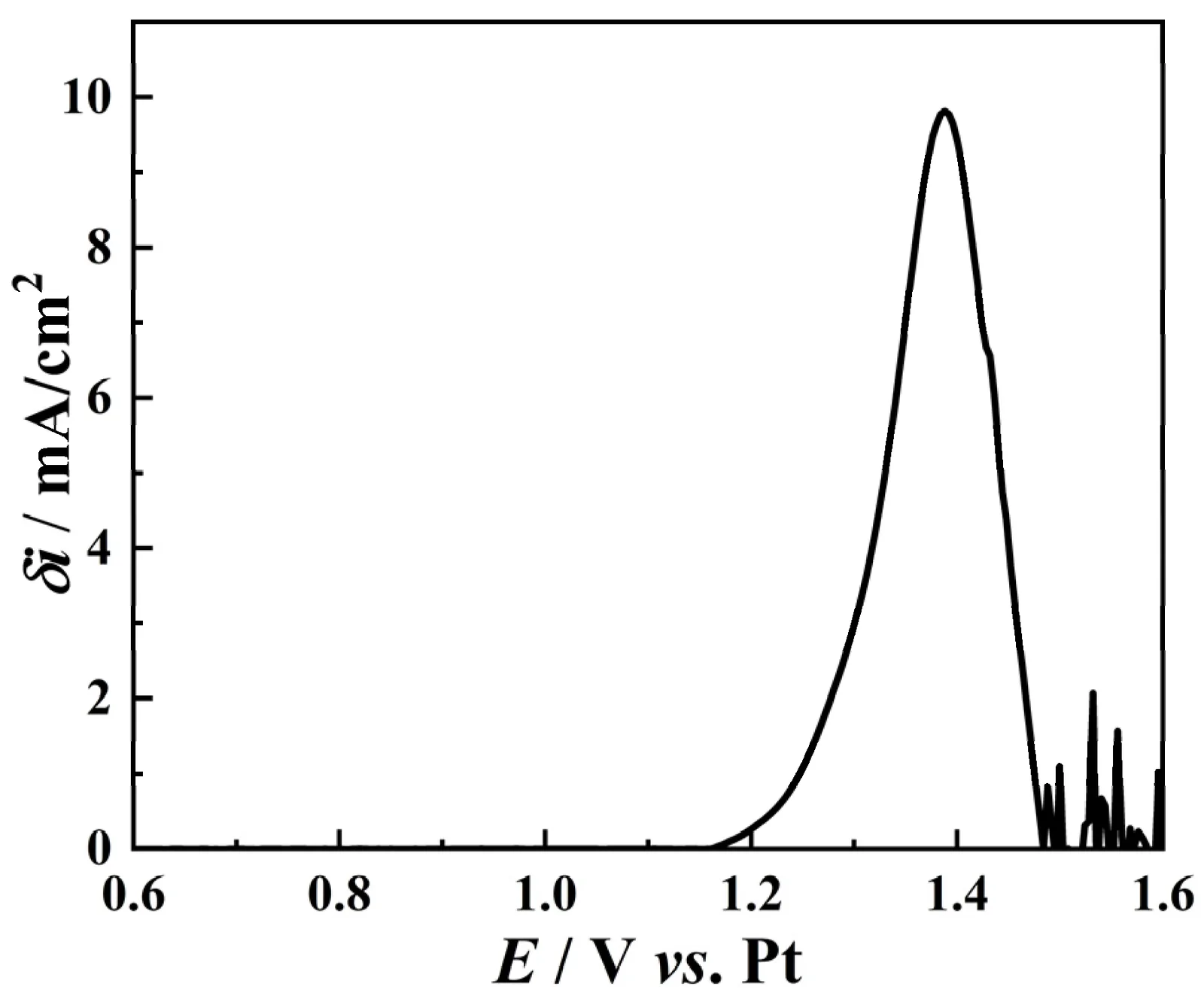
Square wave voltammogram of a FLiBe molten salt without any thermal treatment with unknown oxide concentration. *T* = 600 °C; scan frequency: 10 Hz; pulse height: 25 mV; step potential: 4 mV; working electrode: Au; reference electrode: Pt; auxiliary electrode: graphite.

SWV was carried out for an unpurified FLiBe molten salt with an unknown oxide content. An example of a square wave voltammogram of a FLiBe medium obtained after the fusion without any thermal treatment to remove residual oxide ions at 600 °C with a signal frequency of 10 Hz is shown in Figure 16. This figure exhibits the oxide ions oxidation peak at about 1.35 V vs. Pt with a peak current density of 9.770 mA/cm^2^. Thus, the O^2−^ concentration of this FLiBe melt was calculated to be 6.73 × 10^−2^ mol/kg using Equation (11). For comparison, the same FLiBe molten salt was sampled and transferred for LECO-IC analysis, and the O^2−^ concentration was determined as 6.38 × 10^−2^ mol/kg. The result obtained from the in situ SWV technology is in good agreement with that obtained by the conventional offline LECO-IC analysis, with a low relative error of only 5.48%, as summarized in Table 7. The above results indicate that the SWV technology for quantitative analysis of O^2−^ is feasible and can be applied to the rapid determination of oxide content for an unknown FLiBe molten salt.

### 3.5. Electrolytic Removal of O^2−^ from FLiBe

**Figure 17:**
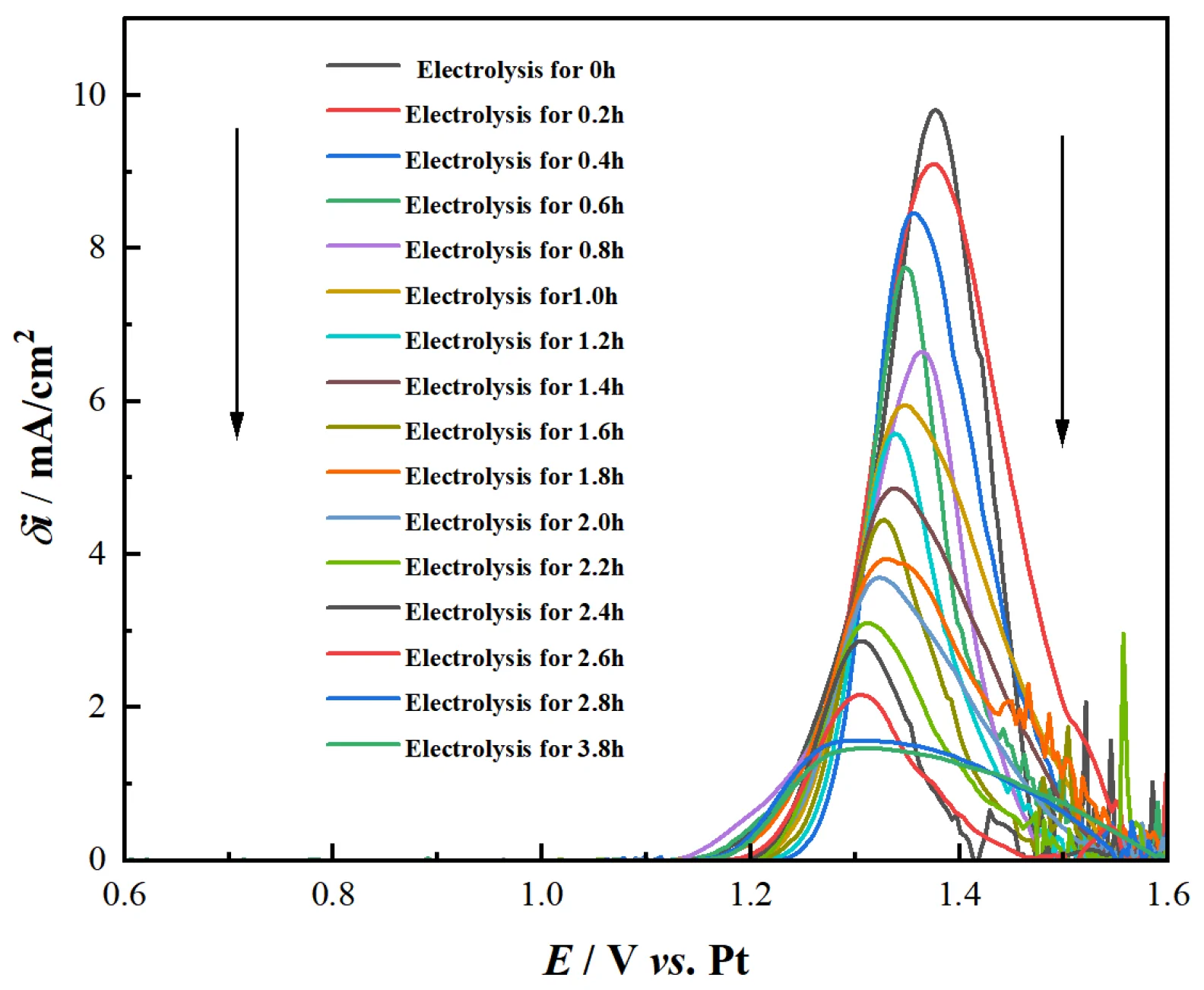
Variation in the square wave voltammograms under different electrolysis durations in the process of electrolytic removal of O^2−^ from FLiBe melts. *T* = 600 °C; scan frequency: 10 Hz; pulse height: 25 mV; step potential: 4 mV; working electrode: Au; reference electrode: Pt; auxiliary electrode: graphite.

**Figure 18:**
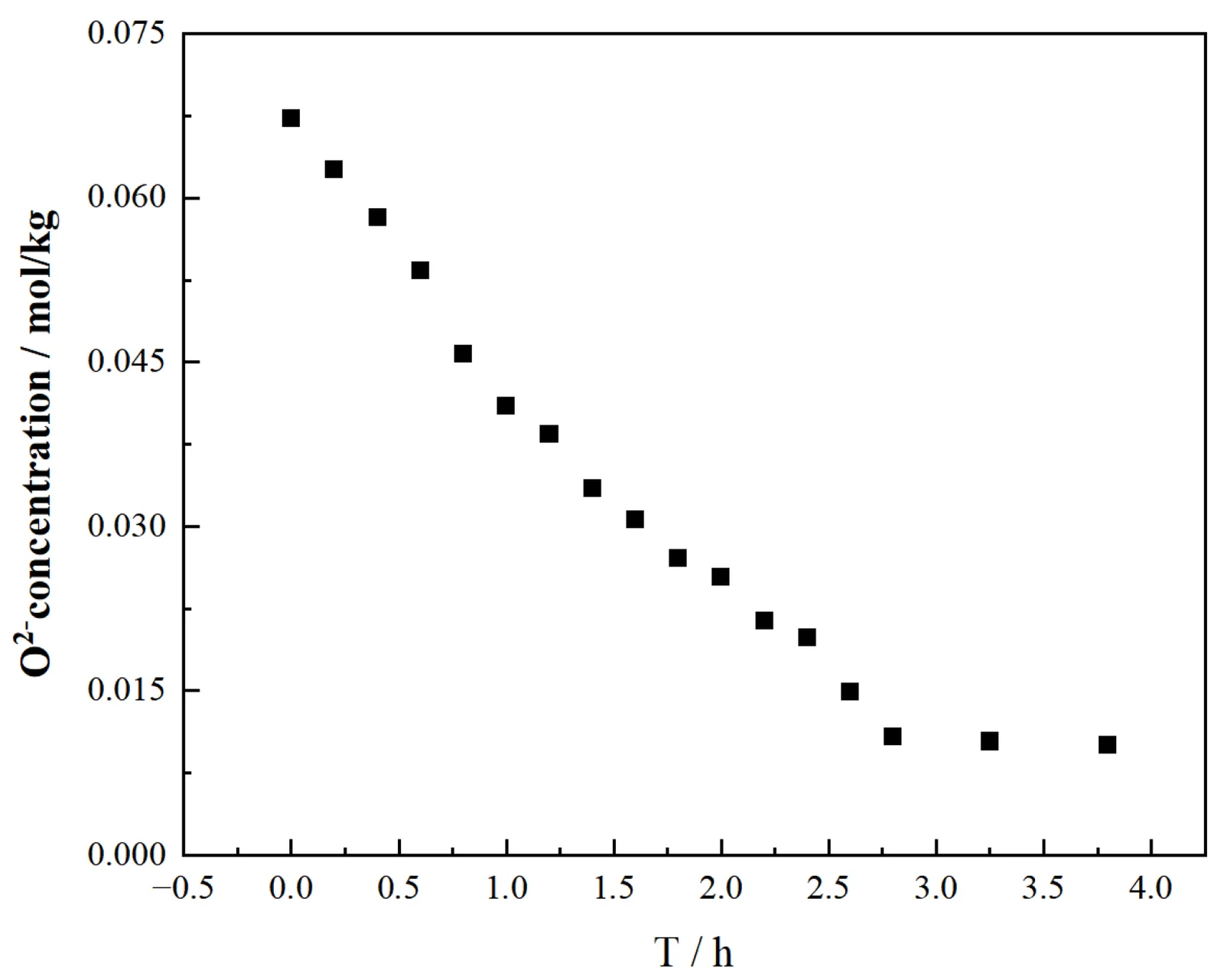
Evolution of residual O^2−^ content in FLiBe molten salt by SWV during potentiostatic electrolysis.

In this study, potentiostatic electrolysis was performed to remove oxide ions in the above unpurified FLiBe molten salt. The electrolysis experiments were conducted at 600 °C using a three-electrode system with a gold working electrode. The electrolytic potential was set at 1.85 V vs. Pt for a total electrolysis duration of 3.8 h. Theoretically, at this potential, O^2−^ is oxidized to O_2_ while ensuring that the gold electrode does not dissolve. In order to monitor the concentration evolution of O^2−^ ions in the electrolysis process, SWVs were conducted at different electrolysis durations. During the early stage of electrolysis, SWV scans were conducted every 0.2 h, while in the later stage, the interval was 1 h. The corresponding square wave voltammograms under different electrolysis times are displayed in Figure 17. The oxidation peak current density at around 1.3 V vs. Pt decreased significantly from the initial 9.770 mA/cm^2^ to 9.097 mA/cm^2^ after 0.2 h of electrolysis. The corresponding O^2−^ concentration was thus decreased from 6.73 × 10^−2^ mol/kg to 6.26 × 10^−2^ mol/kg according to the quantitative equation of SWV (Equation (11)). As the electrolysis proceeds, the peak current density decreases continuously, indicating that O^2−^ is continuously electrolyzed into O_2_(g) and removed. As the electrolysis time increased from 0.2 h to 2.6 h, the oxidation peak current of O^2−^ gradually decreased, and thus the O^2−^ concentration decreased from 6.26 × 10^−2^ mol/kg to 1.49 × 10^−2^ mol/kg. After electrolysis for 2.8 h, the peak current density decreased to 1.562 mA/cm^2^; the corresponding concentration of O^2−^ at this moment is 1.08 × 10^−2^ mol/kg according to Equation (11). After this, the removal rate of O^2−^ ions slowed down. Until 3.8 h, the peak current density decreased from 1.562 mA/cm^2^ to 1.463 mA/cm^2^. That is, during the last hour of the electrolysis process, the O^2−^ concentration decreased only from 1.08 × 10^−2^ to 1.01 × 10^−2^ mol/kg. This result indicates that at low O^2−^ concentrations, the electrolytic removal of oxide ions becomes difficult. The variation trend of residual O^2−^ concentration as a function of electrolysis time is summarized in Figure 18.

For comparison, molten salt samples collected at five different electrolysis durations (0 h, 0.8 h, 1.8 h, 2.8 h, and 3.8 h) were analyzed by offline LECO-IC analysis, and the oxide variation results are shown in Table 8. The SWV results are highly consistent with those obtained by LECO-IC, with relative errors within ±6%, demonstrating the reliability of SWV quantitative technology.

The electrolytic removal efficiency was calculated based on the O^2−^ concentration difference before and after electrolysis using Equation (14). The results show that the potentiostatic electrolysis reduces the O^2−^ concentration from the initial 6.73 × 10^−2^ mol/kg to 1.01 × 10^−2^ mol/kg, achieving a deoxygenation efficiency of 85%. Although the electrolysis removal process of O^2−^ seems to become difficult under low concentration conditions (between 2.8 h and 3.8 h), by means of potentiostatic electrolysis, the concentration of O^2−^ was successfully reduced to 1.01 × 10^−2^ mol/kg, corresponding to 162 ppm, which met the TMSR-LF1 requirement that the O^2−^ content in the fuel salt should be lower than 200 ppm. This work validates the feasibility of potentiostatic electrolysis for oxide removal from FLiBe molten salt.

(14) $$\eta =\frac{C_{0}-C_{f}}{C_{0}}\;\times \;100\%$$

where *η* is the electrolytic removal efficiency of O^2−^, *C*_0_ is the initial O^2−^ concentration before electrolysis, and *C_f_* is the final O^2−^ concentration after electrolysis.

## 4. Conclusions

In this work, a three-electrode system with a gold working electrode was adopted to systematically investigate the electrochemical behavior and electrolysis removal of O^2−^ in FLiBe molten salt. Then, an in situ electrochemical quantitative analysis method for O^2−^ was established. Based on this, a quick determination and monitoring of O^2−^ concentration in FLiBe was realized. Furthermore, electrochemical removal of O^2−^ from FLiBe molten salt was performed and achieved a relatively high extraction efficiency. This study offers reliable theoretical and technical support for the in situ analysis and online monitoring of O^2−^ impurity content in FLiBe molten salt. Meanwhile, this paper proposes an innovative route for oxide removal of FLiBe using molten salt electrolysis technology.

The main conclusions are summarized as follows:

(1) Electrochemical behavior of O^2−^ in FLiBe molten salt. CV, SWV, and CP were employed to characterize the electrochemical behavior of O^2−^. Comparative analysis indicates that SWV is more suitable than CV for studying the electrochemical behavior of O^2−^. The SWV results confirm that O^2−^ is oxidized to O_2_ at 1.3 V vs. Pt with the oxidation mechanism of O^2−^ = 1/2O_2_(g) + 2e^−^, and the electrode reaction is controlled by ion diffusion. The CP results revealed a plateau of 1.3 V vs. Pt for O^2−^/O_2_ oxidation and proved that the electrochemical behavior of O^2−^ complies with the Sand law, where the value of *Iτ*^1/2^ remains constant. The CP results are highly consistent with the SWV findings. The diffusion coefficients *D* of O^2−^ in FLiBe at different temperatures were determined to range from 8.55 × 10^−10^ cm^2^/s to 6.44 × 10^−9^ cm^2^/s within 500–650 °C. The temperature dependence of the diffusion coefficient obeys Arrhenius’ law, and the activation energy *Ea* for O^2−^ diffusion was calculated to be 79.5 kJ/mol.

(2) Establishment of an in situ electrochemical quantitative analysis method for O^2−^. SWV was performed on the FLiBe system with different O^2−^ concentrations. Then, a well-fitted linear relationship between O^2−^ concentration and peak current density was established, and the equation of the straight line is *δi*_p_(mA/cm^2^) = 145.22 [O^2−^](mol/kg). This equation can be applied to the quick determination and monitoring of oxide content for an unknown FLiBe molten salt. The O^2−^ concentration data by SWV electrochemical technology are in excellent agreement with offline results obtained by the combined LECO-IC method, with a relative error within ±6%.

(3) Electrochemical removal of O^2−^ from FLiBe molten salt. Potentiostatic electrolysis technology was adopted for deoxygenation in FLiBe-O^2−^ molten salt at 1.85 V vs. Pt for 3.8 h. During the electrolysis process, SWV was used for online monitoring of the variation in O^2−^ concentration. The O^2−^ concentration was reduced from the initial 6.73 × 10^−2^ mol/kg to 1.01 × 10^−2^ mol/kg, achieving a high oxide removal efficiency of 85%. Meanwhile, the results indicate that under the low oxide content conditions in the later stage of electrolysis, the elimination speed of O^2−^ decreases and the electrolytic process removal of O^2−^ becomes difficult. Nevertheless, the concentration of oxide ions after electrolysis has been successfully reduced to 1.01 × 10^−2^ (162 ppm), which is below the oxide content standard for the TMSR-LF1 fuel salt (200 ppm). The experimental results verify the feasibility of potentiostatic electrolysis for eliminating O^2−^ impurities in fluoride molten salts.

This work presents three key innovations:

(1) This study fills the gap in the fundamental electrochemical parameters of O^2−^ in the FLiBe molten salt, including peak potential, peak current, transferred electron numbers, electrode process nature, diffusion coefficient, activation energy, and electrode reaction mechanism.

(2) The linear quantitative relationship between the SWV peak current density and the O^2−^ concentration was obtained. The established in situ electrochemical method enables quick determination and real-time monitoring of O^2−^ content, effectively overcoming the drawbacks of time consumption, complicated pretreatment, and data hysteresis inherent to conventional offline analysis techniques. Relevant results provide a reference for the online oxide measurement of MSR fuel salt.

(3) This study verified the feasibility of O^2−^ removal through potentiostatic electrolysis and achieved satisfactory removal efficiency. The validated high-efficiency electrochemical deoxygenation strategy provides a novel technical route for fluoride molten salt purification.

This work advances the research on oxide monitoring and purification by establishing a complete electrochemical analysis and deoxygenation system for FLiBe molten salt, which is lacking in previous beryllium-free molten salt studies. Nevertheless, several limitations remain. The electrolytic deoxygenation efficiency decreases significantly at low O^2−^ concentrations, and the effects of trace impurities and long-term high-temperature molten salt corrosion on electrochemical detection and electrolysis performance remain unclear. Furthermore, all experiments were performed under static laboratory conditions, ignoring the flow and irradiation environments of practical MSR operation. Future studies will focus on optimizing electrolytic parameters to improve the removal efficiency of O^2−^ at low-concentration conditions. Additionally, further research can explore the coupled effects of irradiation and hydrodynamic conditions on O^2−^ electrochemical behaviors, and develop reliable in situ monitoring and long-term stable deoxygenation technologies adapted to actual reactor operating conditions.

## Figures and Tables

**Table 1:** O^2−^ concentrations in FLiBe molten salt with different Li_2_O additions.

Added Li_2_O (g)	O^2−^ Concentration Increment Calculated by Equation (4) (mol/kg)	O^2−^ Concentration in Initial FLiBe (mol/kg)	Actual O^2−^ Concentration in FLiBe (mol/kg)
0	0	1.24 × 10^−2^	1.24 × 10^−2^
0.0089	7.42 × 10^−3^		1.98 × 10^−2^
0.0095	7.92 × 10^−3^		2.03 × 10^−2^
0.0144	1.20 × 10^−2^		2.44 × 10^−2^
0.0176	1.47 × 10^−2^		2.71 × 10^−2^
0.0221	1.84 × 10^−2^		3.08 × 10^−2^
0.0253	2.11 × 10^−2^		3.35 × 10^−2^
0.0314	2.62 × 10^−2^		3.86 × 10^−2^
0.0593	4.94 × 10^−2^		6.18 × 10^−2^

**Table 2:** Oxide content analysis results of the FLiBe molten salt used in this study.

Total Oxide Concentration ([O]_LECO_)/mol/kg	Oxygenated Anions Concentration/mol/kg		Oxide in Oxygenated Anions [O]_IC_ Analyzed by IC/mol/kg	O^2−^ Concentration ([O^2−^])/mol/kg
1.42 × 10^−2^	NO_3_^−^	3.06× 10^−4^	1.84 × 10^−3^	1.24 × 10^−2^
	SO_4_^2−^	1.25 × 10^−4^		
	PO_4_^3−^	1.05 × 10^−4^		

**Table 3:** Half peak widths *W*_1/2_ and electron transfer numbers *n* of the FLiBe–O^2−^ (6.18 ×10^−2^ mol/kg) molten salt system at different frequencies measured by SWV (*T* = 600 °C).

Scan Frequency *f* (Hz)	Half-Peak Width *W*_1/2_ (V)	Electron Transfer Number *n*
10	0.116	2.28
20	0.125	2.12
30	0.125	2.12
40	0.140	1.89

**Table 4:** Comparison of O^2−^ diffusion coefficients in various fluoride molten salts at different temperatures.

T/°C	500	550	600	650
D/cm^2^/s (FLiBe) (this study)	8.55 × 10^−10^	1.38 × 10^−9^	2.74 × 10^−9^	6.44 × 10^−9^
D/cm^2^/s (LiF–NaF) [28]	5.49× 10^−7^	1.66 × 10^−6^	4.35 × 10^−6^	1.04 × 10^−5^
D/cm^2^/s (FLiNaK) [35]	7.29 × 10^−7^	1.21 × 10^−6^	1.76 × 10^−6^	3.06 × 10^−6^

**Table 5:** Peak current densities of O^2−^ measured by SWV in the FLiBe molten salt containing different O^2−^ concentrations at 10 Hz and 600 °C.

O^2−^ Concentration in Molten Salt (mol/kg)	O^2−^ Peak Current Density from SWV (mA/cm^2^)
1.24 × 10^−2^	1.740
1.98 × 10^−2^	2.545
2.03 × 10^−2^	3.003
2.44 × 10^−2^	3.608
2.71 × 10^−2^	4.186
3.08 × 10^−2^	4.526
3.35 × 10^−2^	4.750

**Table 6:** The potential variations at different O^2−^ concentrations at 600 °C.

O^2−^ Concentration (mol/kg)	O^2−^ Peak Potential by SWV (V)	O^2−^ Peak Potential Difference with E_1_ by SWV (V)	O^2−^/O_2_ Potential Difference with E_1_ Calculated by Nernst Equation (V)	Potential Drift of Pt Quasi Reference Electrode (V)
1.24 × 10^−2^	1.262	0	0	0
1.98 × 10^−2^	1.274	0.012	−0.018	0.030
2.03 × 10^−2^	1.280	0.018	−0.019	0.037
2.44 × 10^−2^	1.299	0.037	−0.025	0.062
2.71 × 10^−2^	1.304	0.042	−0.029	0.071
3.08 × 10^−2^	1.296	0.034	−0.034	0.068
3.35 × 10^−2^	1.302	0.040	−0.037	0.077

**Table 7:** Comparison of O^2−^ content in FLiBe without thermal treatment determined by SWV and LECO-IC.

O^2−^ Oxidation Peak Current Density (mA/cm^2^)	O^2−^ Content Calculated by SWV Using Equation (11)	O^2−^ Content Measured by LECO-IC	Relative Error (%)
9.770	6.73 × 10^−2^ mol/kg	6.38 × 10^−2^ mol/kg	5.48

**Table 8:** The concentration variations in O^2−^ during the oxide removal process of FLiBe molten salt electrolysis: Comparison of SWV and LECO-IC test results.

Electrolysis Time t/h	O^2−^ Oxidation Peak Current Density (mA/cm^2^)	O^2−^ Content Calculated by SWV Using Equation (11) (mol/kg)	O^2−^ Content Measured by LECO-IC (mol/kg)	Relative Error (%)
0	9.770	6.73 × 10^−2^	6.38 × 10^−2^	5.48
0.8	6.646	4.58 × 10^−2^	4.69 × 10^−2^	−2.34
1.8	3.937	2.71 × 10^−2^	2.66 × 10^−2^	1.88
2.8	1.562	1.08 × 10^−2^	1.14 × 10^−2^	−5.26
3.8	1.463	1.01 × 10^−2^	1.05 × 10^−2^	−3.81

## Data Availability

The original contributions presented in this study are included in the article. Further inquiries can be directed to the corresponding author.
